# MicroRNAs and Toll-like Receptor/Interleukin-1 Receptor Signaling

**DOI:** 10.1186/1756-8722-5-66

**Published:** 2012-10-18

**Authors:** Anthony Virtue, Hong Wang, Xiao-feng Yang

**Affiliations:** 1 Cardiovascular Research Center and Department of Pharmacology, Temple University School of Medicine, 3500 North Broad Street, MERB 1059, Philadelphia, PA, 19140, USA

**Keywords:** MicroRNAs, Toll-like receptors, Interleukin-1 receptor, mRNA stability, Inflammation

## Abstract

The discovery of miRNAs has revolutionized the way we examine the genome, RNA products, and the regulation of transcription and translation. Their ability to modulate protein expression through mRNA degradation and translation repression resulted in avid scientific interest in miRNAs over the past decade. This research has led to findings that indicate miRNAs can regulate an array of cellular functions such as cellular apoptosis, proliferation, differentiation, and metabolism. Specifically, the capability of miRNAs to finely-tune gene expression naturally lends itself to immune system regulation which requires precise control for proper activity. In fact, abnormal miRNAs expression is often seen with inflammatory disorders like rheumatoid arthritis, systemic lupus erthematosus, experimental autoimmune encephalomyelitis, and inflammatory cancers. As a result, research investigating miRNAs modulation of immune cell proliferation, differentiation, and cellular signaling has yielded fruitful results. Specifically, in this review, we will examine the impact of miRNAs on toll-like receptor (TLRs) and interleukin-1β (IL-1β) signaling, which are integral in the proper functioning of the innate immune system. These signaling pathways share several key downstream signaling adaptors and therefore produce similar downstream effects such as the production of pro-inflammatory cytokines, chemokines, and interferons. This review will examine in depth the specific interactions of miRNAs with receptors, adaptor molecules, and regulator molecules within these cellular pathways. In addition, we will discuss the modulation of miRNAs’ expression by TLR and IL-1R signaling through positive and negative feedback loops.

## Introduction

The existence of a molecular system capable of orchestrating the expression of numerous proteins was postulated by researchers for some time; the discovery of microRNAs (miRNAs) confirmed this hypothesis. This breakthrough revolutionized the way we examine the genome organization, RNA products, as well as mRNAs translational and degradation. Originally discovered in 1993, it soon became evident that miRNAs are abundant and often conserved across species. Even more striking is their ability to modulate a wide range of cellular functions, such as apoptosis, proliferation, differentiation, and metabolism. In addition to cellular activities, miRNAs were also found to be capable of regulating a broad spectrum of systemic functions like inflammatory responses. The nature of miRNAs function, to finely-tune protein expression through mRNAs degradation and translation repression, naturally lends itself to immune system regulation which requires precise control for proper activity. Therefore, it is not coincidence that altered miRNAs expression is often associated with progression and remission of inflammatory disorders like rheumatoid arthritis, systemic lupus erythematosus, and experimental autoimmune encephalomyelitis. Ensuing research investigating miRNAs modulation of immune cell proliferation, differentiation, activity, and cellular signaling has yielded fruitful results. Specifically, in this review, we will examine the impact of miRNAs on the integral toll-like receptor (TLR) and interleukin-1 receptor (IL-1R) inflammatory signaling pathways. Both pathways share several key signaling adaptors and therefore induce similar cellular responses when activated. Together they play an indispensable role in the innate immune system by triggering the production of pro-inflammatory proteins like cytokines, chemokines, and interferons. The specific interactions of miRNAs with receptors, adaptor molecules, and regulator molecules within these cellular pathways will be examined in depth. Finally, the modulation of miRNAs expression by TLR and IL-1R signaling will be reviewed.

## miRNAs

Prior to recent breakthroughs, it was believed that the majority of DNAs were transcribed to mRNAs and then translated into proteins. The remaining RNAs not coding for protein were either ribosomal RNAs, transfer RNAs, or perceived as “junk” with little to no pertinent function. In 2003, at the completion of the Human Genome Project, between 20,000 – 25,000 protein coding genes were identified from the 3 billion nucleotide base-pairs of the human genome. Puzzling was the fact that protein coding sequences only accounted for approximately 2% of the total genome [1]. These findings made it hard to believe that such a large majority of the genome was “junk”. Partial enlightenment came with the discovery of additional classes of non-coding RNAs (ncRNAs). Defined simply, ncRNAs are any RNAs that are not translated into protein. Functional applications of these once over looked segments of RNAs include but are not limited to cell metabolism, cell proliferation, cell differentiation, protein secretion, and embryonic development [2-5]. Several classes of ncRNAs exist including transfer RNAs (tRNAs), small nuclear RNAs (snRNAs), ribosomal RNAs (rRNAs), small interfering RNAs (siRNAs), and microRNAs (miRNAs). Possibly the most intriguing class of ncRNAs is miRNAs whose discovery dramatically revised the dogma that RNAs simply act as a template for protein translation. Mature miRNAs are capable of post-transcriptional gene silencing through base-pair interactions with mRNAs and therefore can regulate protein expression. Thus far, over 1,000 miRNAs have been identified which are predicted to regulate up to 30% of protein encoding genes.

**Figure 1:**
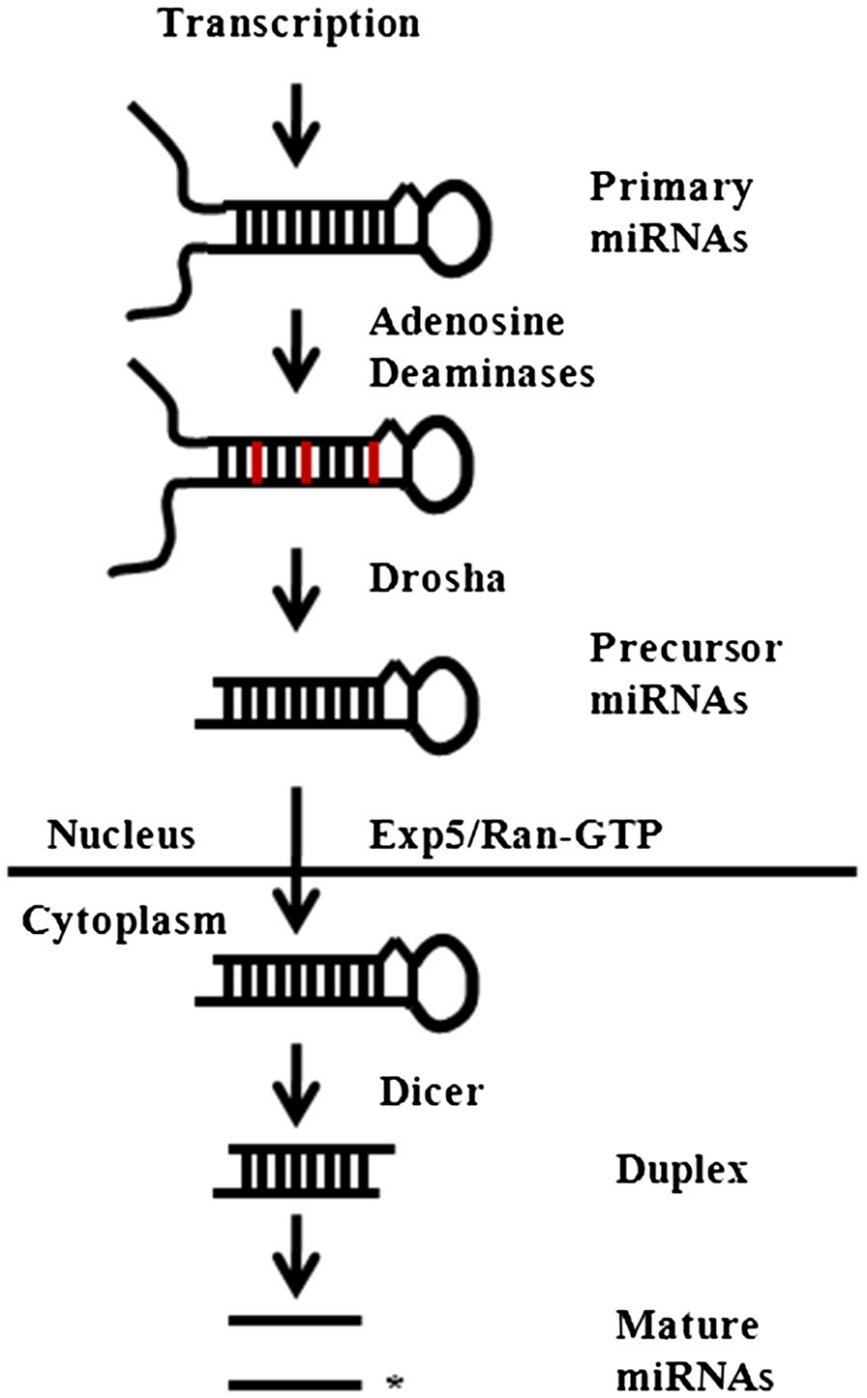
**miRNAs processing and maturation.** Transcribed into a primary transcript of several hundred base-pairs in length, primary miRNAs may undergo editing by adenosine deaminases. Following this, the miRNAs transcript is cleaved by the RNase II enzyme Drosha and then exported to the cytoplasm from the nucleus. Within the cytoplasm the precursor miRNAs transcript is further cleaved by the RNase-III enzyme Dicer. The resulting duplex is then unwound by RNA helicases resulting in mature miRNAs production.

From a biogenesis standpoint, miRNAs are transcribed in similar fashion to mRNAs encoded by protein coding genes. Their genomic sequence can be found within introns of other genes or can be encoded independently. Once transcribed into primary miRNAs, which can be several hundred base-pairs in length, the transcripts may undergo editing by adenosine deaminases. This post-transcriptional modification leads to the exchange of adenosine with inosine which results in an alteration to the transcript’s base-pairing, potentially leading to structural changes. After this, the transcripts undergo processing by the RNase II enzyme Drosha within the nucleus. Cleaved to a length of approximately 70 base-pairs, these precursor miRNAs are then exported from the nucleus to the cytoplasm by exportin 5 and Ran-GTP. Within the cytosol the precursor miRNAs are further cleaved by the RNase-III processing enzyme Dicer to their mature length of 18–23 base-pairs. Upon dicer cleavage, duplexes formed by the hairpin structure of the precursor miRNAs are unwound by RNA helicases (Figure 1). A single strand then enters the RNA-induced silencing complex (RISC), which then facilitates miRNAs-directed mRNAs translation repression or cleavage [6,7].

miRNAs:mRNAs interactions usually occur at the 3’ untranslated region (3’UTR) of mRNAs through imperfect Watson and Crick base-pairing. Noteworthy is that imperfect pairing facilitates the possibility of multiple binding sites for a single miRNA with a particular mRNA. It also promotes promiscuity allowing for multiple mRNA partners for a single miRNA. However, the molecular details of how miRNAs specifically target mRNAs temporally and dynamically are still unknown. Despite this, several structural features which promote miRNAs:mRNAs binding have been elucidated. It has been determined that a region of 6–8 nucleotides, at residues 2–7 on the 5’ end of miRNAs, is critical in establishing miRNAs:mRNAs interactions and is commonly referred to as the “seed region” [8]. The significance of this region is supported by the high level of conservation at the 5’ ends of related miRNAs [9-12]. Meanwhile, the 3’ ends of miRNAs have been demonstrated to participate in base-pairing as well. With the ability to contribute to mRNAs interference, it is postulated that while the 5’ end is required for target identification, the 3’ end is responsible for modulating repression strength [8,9,13]. Specifically, additional binding in the 3’ end of miRNAs with emphasis on nucleotides 12–17 demonstrated augmented miRNAs repression [8]. Other structural features have also been accredited with facilitating miRNAs efficacy. For example, AU richness proximally flanking the seed region binding site has been shown to contribute to miRNAs efficacy, although these contributions quickly diminish with distance from the binding site [8,14,15]. In addition, the location of the miRNAs binding site within the 3’ UTR of mRNAs also plays a role in determining miRNAs functionality. Sites situated near either end of the 3’UTR of mRNAs have been reported to demonstrate greater activity compared to sites that reside in the center of the 3’UTR region of mRNAs. It should be noted that this effect was more pronounced in longer 3’UTRs and that site-conservation in these areas is greater than centrally located sequences. However, sites located to close the open reading frame or within 15 nucleotides of the stop codon have been found to have low binding affinity [8,11,16,17]. Aside from these structural features, it has also been revealed that proximal miRNAs binding sites tend to interact synergistically while multiple sites at distance behave independently with an additive affect [8,9,13,18,19]. This synergistic affect potentially allows for efficient protein regulation with minimal increase in miRNAs cellular levels.

**Figure 2:**
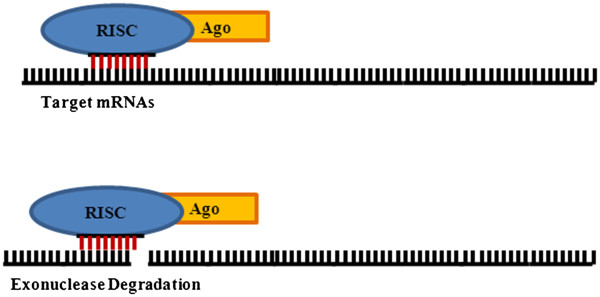
**miRNAs endonucleic cleavage.** Upon miRNAs-directed association of the RNA-induced silencing complex (RISC) with mRNAs, the intrinsic enzymatic protein Argonaute 2 can induce endonucleic cleavage. This is possible as a result of the RNase H activity of the PIWI domain of Argonaute 2. The mRNAs fragments then undergo exonuclease degradation.

As mentioned previously, miRNAs can biologically cause mRNAs cleavage or translation repression. miRNAs-directed endonuclease cleavage of mRNAs is functionally carried out by the highly conserved family of Argonaute proteins [20]. The presence and variety of Argonaute proteins is cell type- and species-specific, allowing for the possible formation of miRNA-specific RISC complexes [21]. In humans, four Argonaute subfamily members exist, although Argonaute 2 (Ago2) is the only member with intrinsic enzymatic activity [22,23]. Approximately 100 kDa in size, Argonaute proteins are basic in nature and contain a PAZ and PIWI domain [24]. The PAZ domain has shown affinity for single- and double-stranded RNAs and therefore allows Argonaute proteins to bind to the stem-loop structure of pri-miRNAs during biogenesis and to mature miRNAs in the RISC complex [25-28]. Meanwhile, the highly conserved PIWI domain contains an RNase H domain which provides certain Argonaute proteins their splicing activity. This theory is supported by the fact that RNase H slicing results in a 3’ overhang which is associated with miRNAs-directed cleavage (Figure 2) [20,29-31]. Slicing typically occurs under conditions of perfect base-pairing between miRNAs and mRNAs although some mismatches can be tolerated [32-34]. It should be noted, that perfect base-pairing does not guarantee endonucleolytic cleavage [35]. This suggests that additional RISC-associated molecules are needed for the cleavage of certain mRNAs. Following cleavage, mRNA fragments undergo standard degradation through either conserved or eukaryotic-specific pathways. Independent of the pathway utilized, initial removal of the 3’poly (A) tail is followed by exonuclease degradation [36,37].

**Figure 3:**
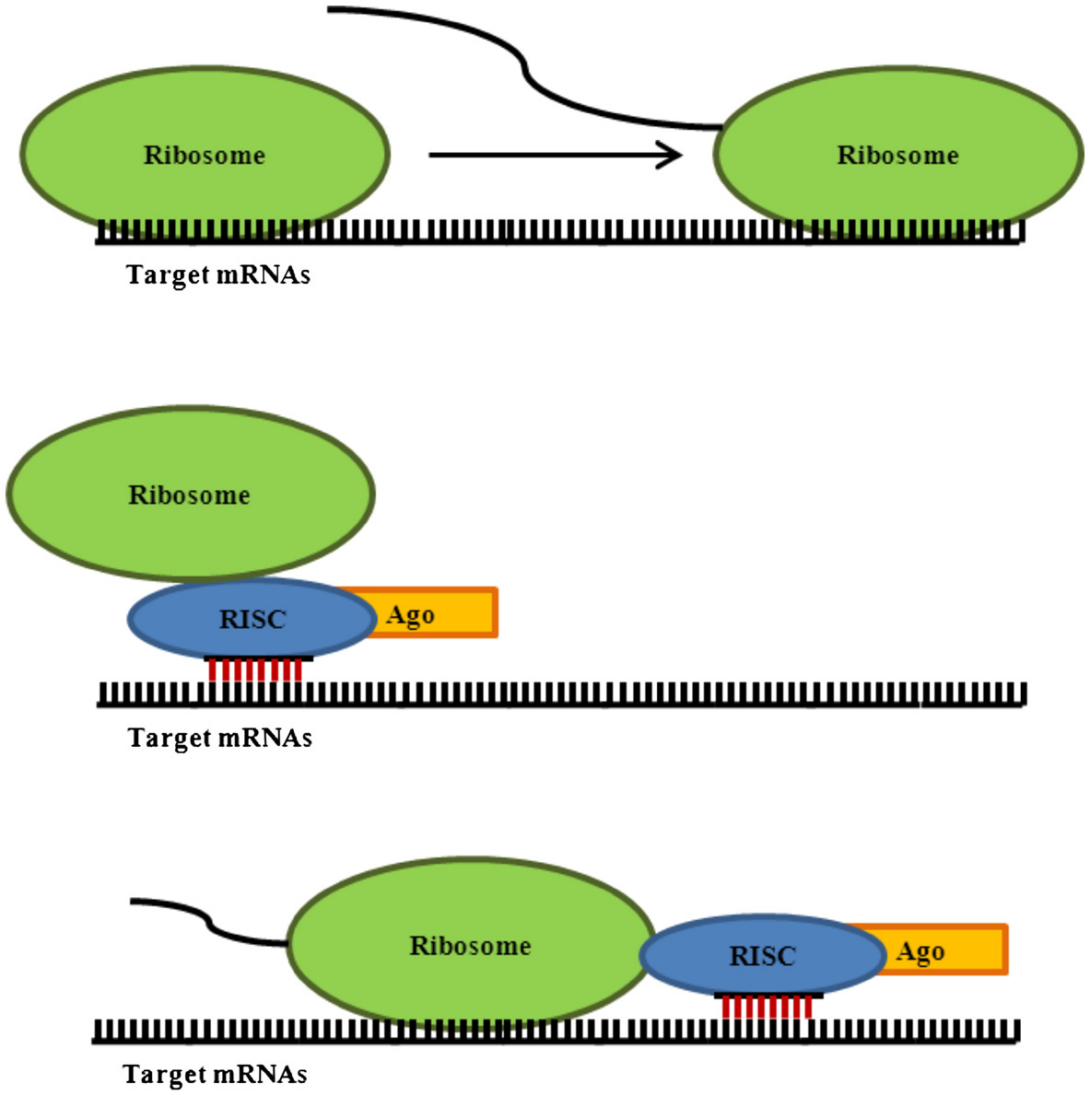
**miRNAs translation repression.** Typically, mRNAs is translated into protein by ribosomes (above). However, miRNAs can inhibit this activity through blockade of translation initiation (middle) or post-initiation translation (below). During translation initiation, repression by RISC proteins prevent the association of ribosomes with mRNAs and thereby translation. With post-initiation translation repression, ribosomes associate with the mRNAs but are incapable of completing translation either due to premature disassociation or physical impediment by associated miRNAs. In addition, post-initiation translation repression may occur due to diminished elongation rates as a result of the presence of miRNAs.

Translational repression is the other mechanism utilized by miRNAs. Described as early as 1993, several studies have been conducted with a range of miRNAs that demonstrate a reduction in protein expression in the midst of unwavering or minimally altered mRNAs levels [35,38-42]. Despite this knowledge, the exact mechanism of how the repression is facilitated is unknown. It is postulated that this suppression is most likely a by-product of an alteration to translation initiation or post-initiation (Figure 3). In fact, adequate amounts of data exist to support both mechanisms [43-50]. It appears that miRNAs which instigate translation initiation repression require the presence of Ago-2 [51,52]. Although the exact biochemical interactions of these proteins have yet to be elucidated, it is known that structurally Ago-2 contains a cap binding-link motif similar to that of eukaryote translation initiation factor 4E (eIF4E), a critical protein in translation initiation [53,54]. This fact, along with additional experimental information, suggests that Ago-2 does in fact interact directly with the cap or indirectly through associated proteins [53]. It is logical to extrapolate that RISC association with the cap, through Ago-2, would dramatically affect translation initiation and thereby repress translation.

Meanwhile, sedimentation studies conducted with mRNAs under miRNAs repression yield results which indicate the association of polysomes. This provides evidence that particular miRNAs function through post-initiation translational interference [48,49,55-58]. Furthermore, the activity of the associated polysomes in translation was verified through puromycin-sensitivity assays [58]. It can be postulated that two mechanisms may be involved in post-initiation suppression. First, miRNAs association could result in elongation interference leading to a dramatic reduction in elongation rate. Second, the presence of miRNAs may lead to premature ribosomal dissociation prior to the completion of translation as a consequence of mRNAs instability or physical impediment. Currently the exact mechanism is unclear, which may indicate the possibility that different miRNAs subtypes exist. Whether initial or post-initial translation repression is utilized, both would account for reductions in protein levels amidst unwavering mRNAs expression witnessed experimentally.

## TLRs

The recent discovery of a class of evolutionarily-conserved receptors has led to the re-examination of the signaling and function of the innate immune system. TLRs are a class of membrane-bound pattern recognition receptors that are capable of identifying particular pathogen associated molecular patterns (PAMPs) and danger associated molecular patterns (DAMPs). Initially discovered in insects, the Toll receptor was determined to play a function in innate immune protection against fungal infections. Pursuit of Toll homologues in humans revealed 11 members, which can identify a wide range of ligands. As Type I integral membrane glycoproteins, TLRs share significant cytoplasmic region homology with interleukin-1 receptors (IL-1R). Within the cytoplasmic tails of the TLRs/IL-1R is a highly conserved region of approximately 200 amino acids referred to as the Toll/IL-1R (TIR) domain [59]. This conserved sequence contains three boxes - box 1 (FDAFISY), box 2 (GYKLC-RD-PG), and box 3 (a conserved W surrounded by basic amino acid residues). Boxes 1 and 2 are believed to be involved in the binding of signaling proteins, whereas box 3 is primarily thought to be involved in the localization of the receptor [59]. Due to this homologous TIR domain, both TLR/IL-1 receptors utilize a shared downstream signaling pathway, which will be discussed in detail later. However, unique extracellular domains allow TLRs/IL-1 receptors to recognize different ligands. The extracellular regions of TLRs contain leucine-rich repeats which form a horseshoe shape and share no homology with IL-1R. Despite the conservation of this region among TLRs, individual receptors maintain the capability of recognizing very different ligands [60]. TLR4 has been attributed with the recognition of several factors including fibrinogen, mouse mammary-tumor virus envelope proteins, taxol, respiratory syncytial virus fusion proteins, heat shock proteins, and lipopolysaccharide (LPS) while TLR3 can identify double-stranded viral DNA [61-68]. The location of ligands recognized by each TLR dictates whether the receptor is located on the cellular membrane or intracellularly. For example, TLR4 ligands are located extracellularly and therefore this particular receptor is found on the cell membrane. In contrast, TLRs −3, -7, and −9 which recognize double-stranded viral DNA, single-stranded RNA, and abnormal nucleic acid motifs respectively, are logically located intracellularly [69-71].

## IL-1

The cytokine interleukin-1 (IL-1) was first discovered in the early 1940s. Produced by active leukocytes and found to induce fever, the protein was aptly named “pyrexin” or “endogenous pyrogen” [72]. It later became evident that this protein did more than induce fever as it mediated an array of biological activities and played a key role in the inflammatory process. Biologically, IL-1 is a potent inducer of chemokines, other cytokines, adhesion molecules, and inflammatory proteins. It soon became evident that the effects of IL-1 were actually the cumulative action of two cytokines, IL-1α and IL-1β [3,4]. Although transcribed from two unique genes found adjacent on chromosome 2, IL-1α and IL-1β share a modest amino acid sequence homology of only 27% [5-8]. Despite this, both have a comparable mature three-dimensional structure [7,8]. This similarity allows both IL-1α and IL-1β to bind to the same receptor, leading to the activation of shared downstream mediators [73]. The 80 kDa receptor, IL-1R1, contains three extracellular immunoglobulin domains and an intracellular domain [31-33]. As mentioned previously, the intracellular portion of IL-1R1 contains a TIR domain and is the reason why it is grouped in the interleukin-1 receptor/Toll-like receptor superfamily. IL-1R1 interaction with IL-1 results in the recruitment of a second receptor chain termed IL-1R accessory protein (IL-1RAcP) [34]. Dimerization of IL-1R with IL-1RAcP results in receptor activation.

## MyD88-dependent signaling pathway

**Figure 4:**
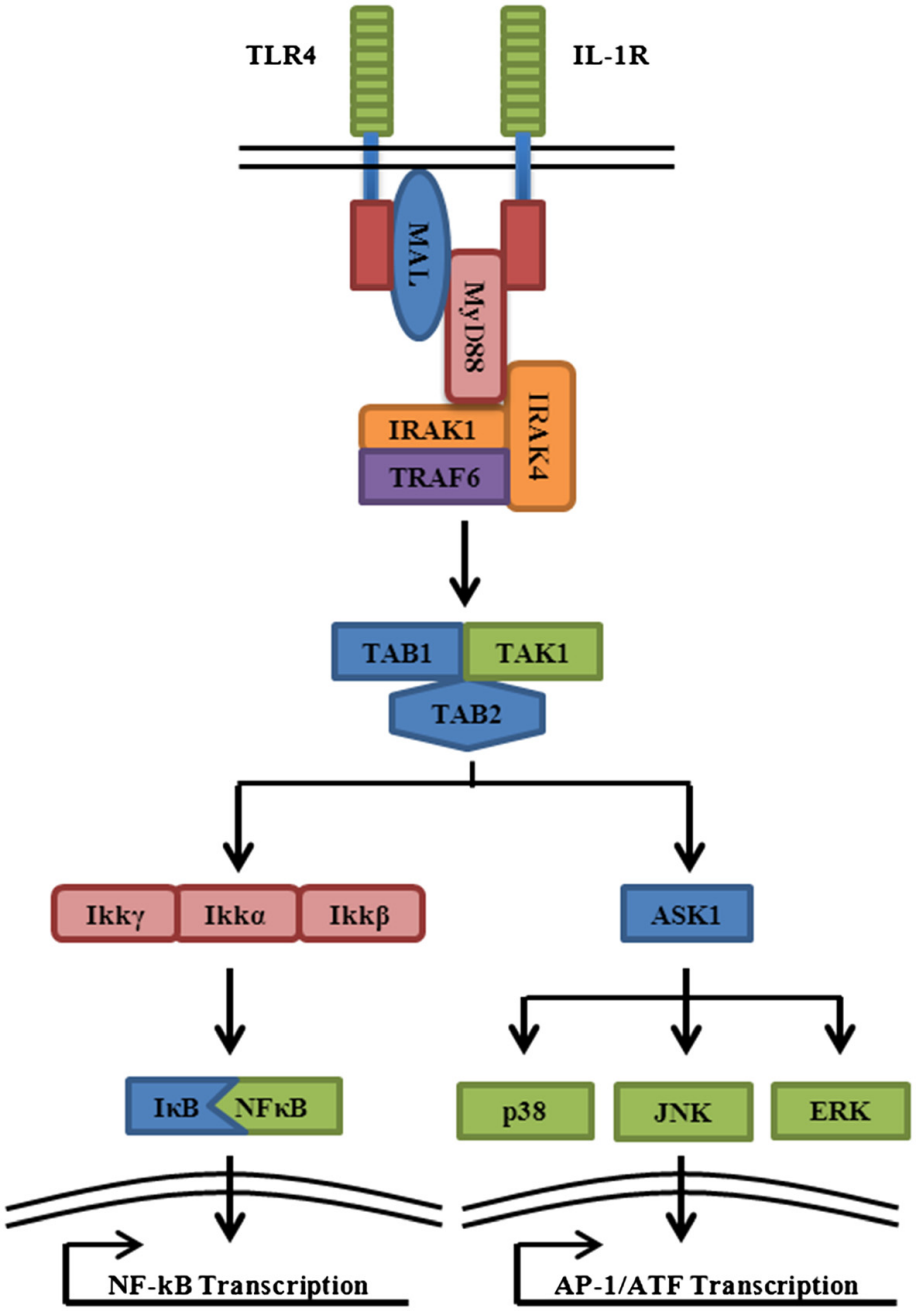
**MyD88-dependent TLR/IL-1R signaling.** TLR/IL-R activation leads to the association of MyD88 (myleoid differentiation primary-response protein 88), which can be enhanced by the presence of MAL (MyD88 adaptor-like) (TLR4 signaling only). As a result, IRAK1 and IRAK4 (interleukin-1 receptor-associated kinase) are recruited to the receptor. Following IRAK1 phosphorylation by IRAK4, TRAF6 (TNF receptor associated factor 6) interacts with IRAK1. This leads to the dissociation and relocation of IRAK1, IRAK4, and TRAF6 to the plasma membrane. There, interaction with the complex of TAK1 (TGF-β-activated kinase 1), TAB1 (TAK1-binding protein), and TAB2 leads to the relocation and association of TAK1 to either the IKK (IκB kinase complex) complex or ASK1 (apoptosis signal-regulating kinase 1). Interface with ASK1 results in downstream MAPK signaling leading to AP-1/ATF transcription factor activity, whereas interaction with IKK leads to IκB degradation and NF-κB transcription factor activity.

Following Toll-like/IL-1 receptor activation conformational changes occur which trigger a shared downstream signaling cascade (Figure 4) [74]. Myeloid differentiation primary-response protein 88 (MyD88) is the first adaptor recruited to the activated receptor complexes. Due to a carboxyl-terminal TIR domain, MyD88 can directly interact with TLRs/IL-1R through TIR-TIR interactions. In fact, mice deficient of MyD88 have displayed an inability to produce IL-6 or tumor necrosis factor-α (TNF-α) in response to exposure with known TLR microbial ligands or IL-1, reaffirming the significance of MyD88 in TLR/IL-1 signaling. It should be noted that in the specific instances of TLR-2 and −4 signaling the association of another TIR-domain containing protein, MyD88 adaptor-like (MAL), facilitates the binding of MyD88. It is believed that the presence of the electro-negative TIR domain of MAL is necessary for the proximal association of the TIR-domains of TLR4 and MyD88, which are both electro-positive [75]. Structurally, MyD88 also contains an N-terminal death domain which facilitates interactions with downstream adaptors [76]. Specifically, IRAK1 and IRAK4, members of the interleukin-1 receptor-associated kinase (IRAK) family, also contain an N-terminal death domain and have a serine/threonine-kinase domain which provides intrinsic kinase function [77,78]. Following TLR/IL-1R activation the intrinsic kinase function of IRAK1 is strongly induced leading to the downstream activation of nuclear factor κB (NF-κB) [77,79]. However, abrogation of IRAK1 kinase activity still results in NF-κB activation, mitigating the necessity of IRAK1 in TLR/IL-1 signaling. Despite this, the deletion of IRAK1 results in reduced cytokine production in response to LPS, a known TLR-4 ligand, and IL-1 [80,81]. This indicates a supporting role of IRAK1 in TLR/IL-1 signaling. The selective deletion of IRAK4 on the other hand, completely alleviates IL-1-mediated NF-kB activity, indicating its necessity for TLR/IL-1 signaling [82]. Interestingly, it has been determined that IRAK1 is a substrate of IRAK4 but not vice versa [83]. Utilizing this information, one can surmise that IRAK4 directly interacts with the adaptor MyD88 and then interacts with IRAK1 leading to IRAK1 phosphorylation. This induces IRAK1’s kinase activity, causing autophosphorylation and leads to a conformational change which makes it suitable for further adaptor interactions [74].

Containing six family members, the TNF receptor associated factors (TRAFs) are a group of evolutionary conserved adaptor molecules [84]. Structurally, TRAFs contain a characteristic coiled-coil domain within the N-terminus while the C-terminus functions as an interface for upstream interactions and self-association [85]. Functioning downstream of the IRAKs is TRAF6 which contains the binding motif P-X-E-X-X that is also found three times within IRAK1. TRAF6 binding to IRAK1 leads to the dissociation of TRAF6, IRAK1, and IRAK4 [86]. This complex proceeds to the plasma membrane where it interacts with the complex of transforming growth factor-β (TGF-β)–activated kinase 1 (TAK1), TGF-β-activated protein kinase 1-binding protein 1 (TAB1), and TGF-β-activated protein kinase 1-binding protein 2 (TAB2). The mitogen-activated protein kinase (MAPK) kinase kinase TAK1 is essential in IL-1 and TNF-α-induced NF-κB activation [87]. TAB1 is capable of boosting the activity of TAK1 while TAB2 acts as a facilitator of TAK1 activation by acting as an adaptor to TAK1/TRAF6 association [88,89]. It should be noted that a TAB2 deficiency does not result in impaired IL-1 and TNF-induced NF-κB activation [90]. This is most likely explained by the discovery of TAB3, which is believed to have TAB2 redundant activity. This hypothesis was validated when transfection with small interference RNA directed against both TAB2 and TAB3 resulted in abrogation of NF-κB activation induced by IL-1 and TNF-α [91]. Once activated, TAK1 proceeds to interface with the inhibitor of NF-κB (IκB) kinase complex (IKK). This interaction leads to IKK activation causing downstream phosphorylation of IκBs resulting in their degradation. As a result, this frees NF-κB, a protein comprised of two subunits p50 and p65, from IκB repression. This allows NF-κB to translocate to nucleus where the transcription augmentation of NF-κB-dependent pro-inflammatory genes occurs.

Aside from NF-κB activation induced by the TLR/IL-1 signaling just discussed, an alternative pathway can be triggered midway through the signaling cascade by both TLRs and IL-1R. This results in the activation of p38 mitogen-activated kinases (p38), c-Jun N-terminal kinases (JNKs), extracellular signal-regulated protein kinases (ERKs), and the two transcription factors - activator protein-1 (AP-1) and activating transcription factor (ATF) [92-94]. While the exact signaling cascade which leads to the activation of these factors has yet to be clearly defined, it is believed that following TRAF6-induced activation, TAK1 associates with apoptosis signal-regulating kinase 1 (ASK1/MAPKKK5) under certain circumstances instead of IKK as mentioned above [95]. In fact, it has been reported that overexpressed ASK1 can inhibit IL-1 induced NF-κB activation by competing with TRAF6 for TAK1 [96]. Moreover, ASK1-deficient mice were shown to be protected from LPS-induced septic shock, demonstrating the significance of the alternative pathway in innate immune responses [97]. Activation of ASK1 leads to the triggering of p38 and JNK signaling pathways [98]. It should be noted that ASK1-deficient mice only display impaired signaling to LPS and not to the TLR-3 and −9 ligands poly(I:C) or CpG-DNA. Furthermore, impaired TLR-4 signaling appeared to be related to p38 signaling and not JNK signaling [97]. Taken together this may indicate TLR specificity or TLR-specific MAPKKK in p38 and JNK signaling.

## MyD88-independent signaling pathway

**Figure 5:**
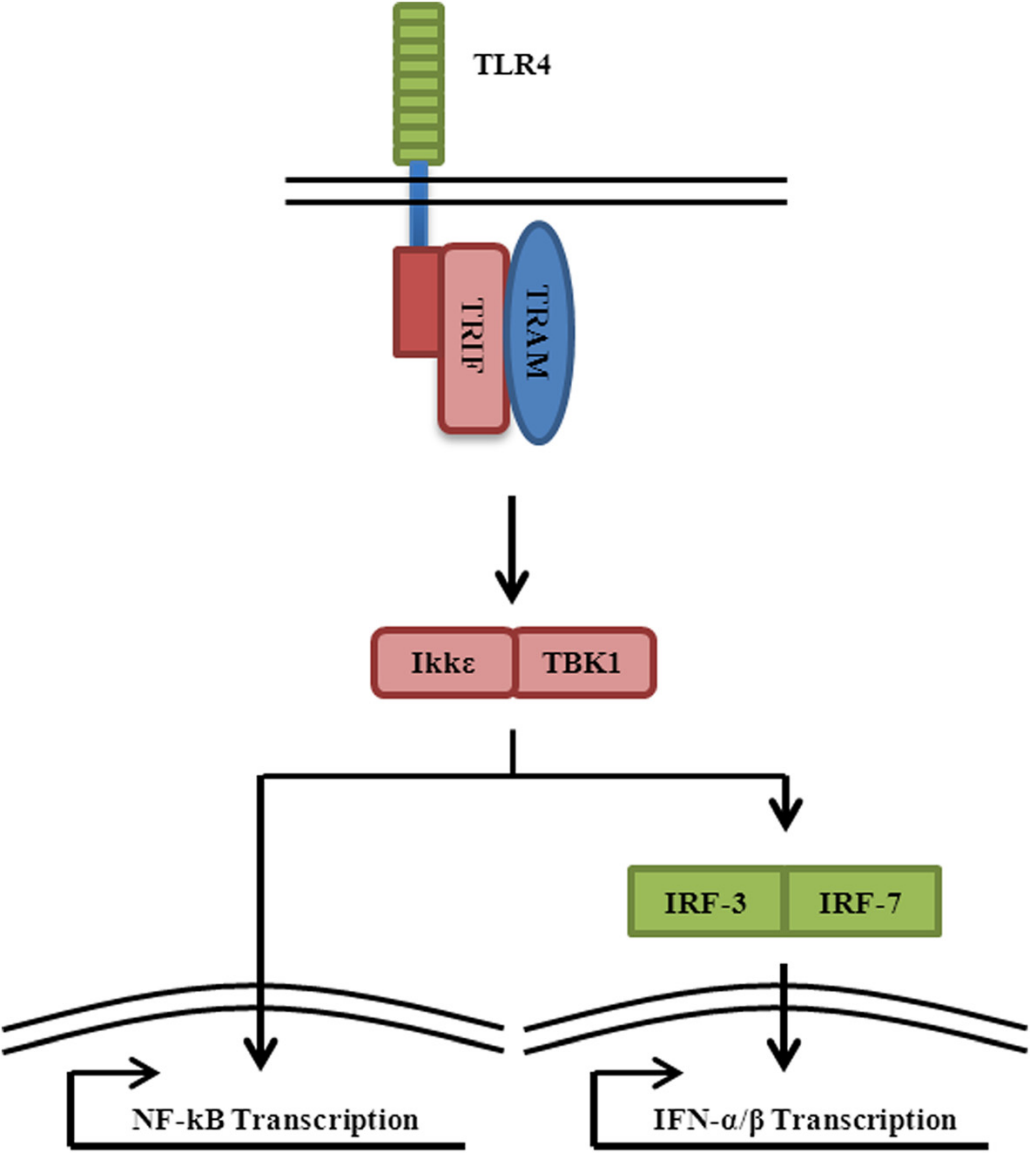
**MyD88-independent TLR signaling.** Particular TLRs, like TLR3 and TLR4, can initiate MyD88-independent signaling. Receptor activation leads to the association of TRIF (TIR-domain-containing adaptor protein inducing IFN-β) protein. Specific to TLR4 signaling, the adaptor protein TRAM (TRIF-related adaptor molecule) also associates with the receptor to facilitate TRIF binding. As a result of TRIF binding, downstream NF-κB-directed transcription occurs along with IFN-α/β production. Although the exact signaling chronology is undetermined, it is known that two non-canonical IKKs (inhibitor of NF-κB kinase complex), IKKε and TBK1 (TANK-binding kinase 1) are activated. As a result, theses IKKs can initiate both NF-κB and IRF-3 (interferon-3) activity. In addition to IRF-3, IRF-7 is also known to be activated in the independent pathway leading to IFN-α/β production.

While mice deficient of MyD88 have displayed an inability to produce IL-6 or TNF-α when exposed to TLR microbial ligands or IL-1, more extensive experimentation with LPS resulted in delayed NF-kB activation and INF-β production. These results indicated the existence of a MyD88-independent pathway (Figure 5). It has been shown that this alternative pathway activates interferon regulatory factor-3 (IRF-3) and −7 (IRF-7), leading to the induction of IFN-α and IFN-β and is also capable of propagating NF-κB activation. While the MyD88-independent pathway is the secondary pathway employed by TLR-4, its activity has been found to act in conjunction with the MyD88-dependent pathway in the production of pro-inflammatory cytokines. Alternatively, the MyD88-independent pathway is the sole signaling pathway utilized by TLR-3. This is logical since TLR-3 recognizes double-stranded RNA often associated with viruses which are more effectively combated by IFN production as compared to NF-κB mediators. It should be noted that IL-1 signaling cannot facilitate this cascade.

Although the MyD88-independent signaling pathway is less elucidated than the MyD88-dependent pathway, some key players have been identified. Required for interfacing with all TLRs is the presence of a TIR-domain within the direct downstream adaptor protein. To identify this protein, researchers relied on bioinformatics which led to the discovery of TIR-domain-containing adaptor protein inducing IFN-β (TRIF). It was quickly established that TRIF mediates the MyD88-independent pathway based on experimental results in TRIF-deficient mice which displayed impaired IFN-β production (IRF3) upon stimulation with TLR-3 and −4 ligands [99]. Signaling downstream of TRIF are two non-canonical IKKs, IKKε and TBK1. Both have been shown to activate NF-κB and IRF-3 [100-105].

Another TIR-domain containing protein, TRIF-related adaptor molecule (TRAM), was also identified by *in silico* studies [75]. Again, knockout mice were utilized to identify the role of TRAM in TLR/IL-1 signaling. Experimentation with IL-1β revealed no impairment of NF-κB activation, JNK activation, or IL-6 production in TRAM knockouts. This result indicated that TRAM was not involved in the shared TLR/IL-1 MyD88-dependent signaling pathway. This conclusion was further supported when TRAM-deficient mice displayed normal immunological responses to ligands for TLR-2, -7, and −9, which all exclusively use the canonical pathway. Interestingly, exploration of the role of TRAM in MyD88-independent signaling revealed that TLR-4 but not TLR-3 production of IFN-β or other IFN-inducible genes were impaired in TRAM-deficient mice [106]. This suggests an exclusivity of TRAM in TLR-4-mediated MyD88-independent signaling. Currently, it is believed that TRAM facilitates TRIF binding with TLR-4 in a similar fashion as MAL in MyD88/TLR-4 association.

Further examination in TRAM knockout mice revealed that TLR-4-mediated NF-κB activation was impaired in a time-dependent fashion. Initial NF-κB activation, 10–20 minutes following LPS treatment, was undeterred while levels were diminished at 60–120 minutes when compared with wild-type controls. Similarly, JNK activity was also found to be limited in a time-dependent manner [106]. These findings indicate a divergent role between MyD88-dependent and –independent signaling pathways. MyD88-dependent signaling induces a rapid immunological response while MyD88-independent signaling triggers a delayed response allowing for immune sustainability. To ensure that the MyD88-dependent and –independent are the only pathways utilized by TLR-4, MyD88/TRIF double knockout mice were bred. In this model LPS-induced NF-κB activation was completely abolished suggesting the absence of any further signaling cascades.

## JAK2/STAT5/IL-6 signaling

IL-6, a known proinflammatory cytokine, is produced in response to challenge with several TLR ligands and IL-1 signaling. However, the exact signaling required for this induction has not been resolved; specifically, the upstream signal transduction. It has been reported that the NF-κB subunit, p50, can increase IL-6 expression. Furthermore, signal transducer and activator of transcription 5 (STAT5) is activated by LPS treatment in mouse monocyte/macrophage RAW cells and peritoneal macrophages. A possible interaction between p50 and STAT5 was examined and confirmed with co-immunoprecipitation assay in African green monkey kidney fibroblast-like COS-7 cells. To determine the mechanism of IL-6 induction, a chromatin immunoprecipitation assay was conducted and showed that both p50 and STAT5 located to the promoter of IL-6. Traditionally, functioning upstream of STATs are Janus kinases (JAKs). In particular, JAK2 expression was found to be augmented within 1–5 minutes following LPS treatment in RAW cells. This finding, along with comparative downstream effects following deletion of either JAK2 or STAT5, indicates that both are involved in this signaling pathway. Investigation of further upstream signaling revealed that JAK2 could not associate individually with either MyD88 or TLR-4 in co-immunoprecipitation assays. However, JAK2 was found to interact with the MyD88/TLR-4 complex. This evidence suggests the existence of an unidentified adaptor molecule that facilitates this interaction. Interestingly, IL-6 has been demonstrated to induce suppressor of cytokine signaling 1 (SOCS1), a known negative regulator of cytokine signaling. The role of SOCS in TLR/IL-1 signaling will be discussed in-depth in the following section [107].

## Endogenous TLR/IL-1 signaling regulators

Hyper-activation of immune signaling pathways can seriously impact overall health, leading to a wide variety of chronic inflammatory and autoimmune illnesses. Each adaptor molecule within the TLR/IL-1 signaling pathway is under stringent control via phosphorylation, physical interactions, conformational changes, as well as ubiquitin and proteasomal degradation. Furthermore, there are regulatory proteins designed to attenuate TLR/IL-1 signaling which will now be discussed.

A family of eight proteins aptly referred to as the suppressors of cytokine signaling (SOCS), primarily function to inhibit cytokine signaling. Specifically, SOCS1 is induced by LPS and CpG-containing DNA, known activators of the TLR/IL-1 signaling [108]. This led researchers to investigate whether SOCS1 played a role in mitigating TLR/IL-1 signaling. Further investigation revealed that SOCS1-deficient mice were hypersensitive to LPS challenge [109,110]. In addition, treatment of SOCS1-deficient mice with CpG DNA and macrophage-activating lipopeptide 2 both resulted in the augmented expression of pro-inflammatory cytokines [109-113]. These findings led investigators to screen for specific SOCS1 targets within the canonical TLR/IL-1 pathway. SOCS1 was found to directly target the p65 subunit of NF-κB leading to its ubiquitination and degradation [114]. It was also found to target and mitigate the activity of MAL resulting in the diminished down-stream activation of NF-κB [115]. Aside from regulating the canonical TLR/IL-1 pathway, it has also been reported that SOCS1 regulates the alternative MyD88-dependent pathway by suppressing ASK1. SOCS1 can also alleviate TLR MyD88-independent signaling as well. As discussed earlier, the activation of MyD88-indepenedent signaling pathway results in the up-regulation of IFN-β. It has been shown that SOCS1 represses downstream IFN-β effects on JAK/STAT signaling [116]. In addition, the JAK2/STAT5/IL-6 signaling pathway has also been shown to be repressed by SOCS1 [107]. The ability of SOCS1 to mitigate the activity of the canonical TLR/IL-1 MyD88-dependent signaling pathway, the alternative MyD88-dependent pathway, downstream MyD88-independent signaling, and IL-6 production indicates the significance of SOCS1 in mediated the innate immune system.

SH2-containing inositol phosphatase, SHIP1, is a protein tyrosine phosphatase and a potent endogenous NF-κB inhibitor. SHIP1 has been shown to negatively regulate several tyrosine kinase-dependent signaling pathways in myeloid cells, B-lymphocytes, and T-lymphocytes [117]. The expression of SHIP1 and its phosphorylation is induced by LPS. This led researchers to evaluate the role of SHIP1 in TLR/IL-1 signaling and the discovery that SHIP1 negatively regulates this signaling [118-120]. In fact, this finding may explain the systemic autoimmunity and severe inflammation observed in SHIP1-deficient mice. In macrophages, TNF-α and IL-6 production was diminished after LPS stimulation with SHIP1 over-expression [119]. This observation was supported by the data gathered from SHIP1 RNA-interfering studies. In addition, SHIP1 was also found to inhibit the activation of MAPKs induced by LPS stimulation.

The immunosuppressive/anti-inflammatory interleukin, IL-10, has also been attributed with mitigating TLR/IL-1 MyD88-dependent signaling but not MyD88-independent signaling. Levels of IL-6 were found to be augmented in the presence of an IL-10R antagonist and diminished with IL-10 treatment following exposure to MyD88-dependent signaling activator CpG-containing DNA. This effect was not seen when treated with PolyI:C, a MyD88-independent signaling ligand. The diminished MyD88-dependent signaling associated with the treatment of IL-10 can be attributed to diminished protein levels of IRAK4 and TRAF6. In fact, IL-10 antagonism led to prolonged protein levels of IRAK4 and TRAF6 following LPS treatment in comparison to controls. This observation appears to be the direct result of increased ubiquitination of IRAK1, IRAK4, and TRAF6 seen with the treatment IL-10. In support of these findings, the effects of IL-10 on MyD88-dependent signaling were abolished with proteasomal inhibition [121]. It should also be noted that the expression of IL-10 can be regulated by the pro-inflammatory protein programmed cell death 4 (PDCD4). In fact, the inhibition of PDCD4 with siRNA resulted in elevated levels of IL-10 following LPS stimulation. Similar observations were also made in PDCD4-deficient mice.

## miRNAs regulation of TLR/IL-1β signaling

Crucial for proper immune system function, TLR/IL-1 signaling is under stringent regulation. This adept regulation is required to ensure proper host defense while mitigating the deleterious effects of the inflammation process. Previously discussed regulators like SOCS1 and SHIP1 can inhibit various aspects of this shared pathway via transcription and post-translational mechanisms, and we will now discuss the fine-tuned regulation of the TLR/IL-1 pathway by miRNAs via miRNAs-directed mRNAs translation repression or cleavage [6,7].

## miRNAs modulation of TLRs/IL-1R

The most obvious way that miRNAs can affect TLR/IL-1 signaling is by altering the expression of the receptors. However, examination of TLRs and IL-1R for highly conserved miRNAs consensus binding regions with target prediction software like TargetScan and PicTar have yielded few results. This may be the result of species-specific regulation of TLRs and IL-1R by poorly conserved sites or it might be that the critical role of TLRs and IL-1R in immunity facilitates the need for tight transcriptional control or the need for constitutive expression. With that said, the regulation of TLR-2 and −4 by miRNAs has been observed. The direct association of miR-105 with TLR2 mRNAs has been witnessed while working in human oral keratinocytes, while the regulation of TLR4 expression by let-7i in human biliary epithelial cells and by let-7e in macrophages has been reported (Table 1) [122-124]. miR-105 association with TLR-2 mRNAs was predicted by bioinformatics studies and the binding site was confirmed using a luciferase reporter assay. Luciferase activity was diminished in the cells co-transfected with TLR-2 3’UTR binding site sequence vectors and miR-105 mimics. This effect was not seen in the cells co-transfected with scrambled vector controls and the mimic, indicating binding site specificity. Furthermore, the presence of miR-105 inhibitors led to elevated TLR-2 expression detected by Western blots [124]. Similar to miR-105, let-7i was demonstrated to directly bind to TLR4 mRNAs detected by a luciferase reporter. Further experimentation with RT-PCR showed that let-7i mimics, inhibitors, and scrambled controls did not alter TLR4 mRNAs levels. This finding suggests that let-7i controls TLR4 protein expression by translation repression and not via TLR4 mRNA degradation. This was further supported when infection with *Cryptosporidium parvum* led to the up-regulation of TLR4 protein expression but not its mRNAs. This alteration in TLR4 protein expression could be diminished or augmented when cells were transfected with either let-7i mimic or inhibitors respectively [123]. Similar results were seen in macrophages with the expression of let-7 [122]. However, unlike with let-7i, it was clearly illustrated that TLR4 mRNAs levels were suppressed after treatment with a let-7e mimic for 24 hours. Furthermore, when the macrophages were treated with a let-7e inhibitor, TLR-4 mRNAs expression was increased by 3.5 folds after 24 hours [122]. These studies reveal the possible mitigation of TLR expression by miRNAs. It should be noted that no miRNAs association with IL-1R mRNAs has been reported thus far.

**Table 1:** miRNAs Targets within the TLR/IL-β Signaling Pathway

**TLR Receptors**		**TLR/IL-1β Signaling Molecules**	
TLR2	miR-105	MyD88	miR-155
TLR4	let-7i	MAL	miR-145
	let-7e	IRAK1	miR-146
		TRAF6	miR-146
**TLR Regulators**		TAK1	miR-10a
SOCS1	miR-155	TAB2	miR-155
SHIP1	miR-155	IKKα	miR-223
IL-10	miR-106a		miR-15a
			miR-16
		IKKβ	miR-199
		IKKε	miR-155
		NF-κB1	miR-9

## miRNAs regulation of TLR/IL-1β adaptor molecules

Despite the limited number of experimentally verified miRNAs that interact with TLR/IL-1β receptors, there are abundant instances of miRNAs targeting TLR/IL-1 adaptor molecules. This encompasses miRNAs that target multiple adaptors within the TLR/IL-1 pathway and those that target individual proteins. As discussed earlier, MyD88 is an integral adaptor molecule in proper TLR/IL-1 signaling, functioning as the signal transducer for IL-1R and all TLRs, except TLR-3. Therefore, it should not be surprising that this prominent signaling protein is targeted by miRNAs. Predicted by TargetScan and experimentally verified with luciferase reporter assays, it has been clearly demonstrated that miR-155 directly regulates the expression of MyD88. Additionally, the transfection of cells with miR-155 mimics revealed that MyD88 protein expression was modulated in a time and dose-dependent manner. To determine the method of miRNAs repression, RT-PCR was performed following mimic treatment. This resulted in no change in MyD88 mRNAs levels, suggesting that miR-155 modulates MyD88 protein expression by mRNAs translation repression rather than mRNAs degradation [125]. The adaptor protein MAL, which facilitates MyD88 interaction with TLR-2 and TLR-4, has also been found to be regulated by miRNAs. Predicted to be targeted by miR-145, this interaction was confirmed by luciferase reporter assays in human embryonic kidney 293 (HEK293) cells. Western blots were then carried out following mimic or inhibitor transfection of bone marrow cells which verified that MAL protein expression was diminished and augmented, respectively. Unfortunately, mRNAs levels were not examined so the exact mechanism of miRNAs repression cannot be cited. Acting downstream and associating with MyD88, IRAK1 is essential to TLR/IL-1 signaling and has been identified as a target of miR-146a/b. Again, this association was predicted utilizing online software and then confirmed with luciferase reporter assays [126]. This interaction was further confirmed by another group with the transfection of miR-146a mimics and inhibitors in macrophages [127]. IRAK4 has yet to have miRNAs association experimentally identified. This may be the result of the fact that it plays a role in, but is not essential to, TLR signaling. Despite this, the possibility of miRNAs interactions should not be ruled out until further investigation is concluded. Functioning downstream of the IRAKs, TRAF6 has been confirmed to be targeted by miR-146. Luciferase reporter assays with TRAF6 3’UTR-containing vectors verified this association. Online prediction algorithms were then used to identify the exact binding site so that a mutated vector could be constructed. The use of this mutant in luciferase reporter assays confirmed binding site specificity. Mitigation of TRAF6 protein expression was then verified by Western blot following mimic and inhibitor transfection [126,127]. Downstream of TRAF6 is TAK1 which functions to activate the IKK complex. The prediction of a conserved miRNA-10a binding site by TargetScan within the 3’UTR of TAK1 was confirmed in HEK293 cells [128]. Furthermore, it was experimentally demonstrated that TAK1 mRNAs and protein was elevated with miR-10a knockdown in human aortic endothelial cells by qRT-PCR and Western blot, respectively. Facilitating the protein-protein interaction of TRAF6 and TAK1 is TAB2. This important adaptor has been identified to be modulated by miR-155. A predicted association by TargetScan, the miR-155:TAB2 interaction was proven through Western blot, qRT-PCR, and luciferase reporter assay results. This repression was supported by the similar findings by another independent research group [129]. The IKK complex is located downstream of TAK1 and contains 3 subunits. Two of those subunits, IKK-α and IKK-β, have been found to be under miRNAs regulation. IKK-α is mediated by miR-223, miR-15a, and miR-16 while IKK-β is targeted by miR-199. The association between IKK-α and all three miRNAs were confirmed individually with luciferase reporter assays by creating constructs mutated for each specific miRNAs binding site. In addition, transfection with mimics for all three miRNAs results in diminished Iκk-α protein levels in human cervical cancer (HeLa) cells and macrophages. As predicted by PicTar, IKK-β contains three putative miR-199 binding sites. A luciferase reporter assay was utilized to confirm this targeting while the use of mimics and inhibitors demonstrated that IKK-β protein levels could be mitigated. Once the IKK complex phosphorylates IκB, NF-κB is freed from repression to translocate to the nucleus. As mentioned earlier, NF-kB contains two subunits p50 and p65. NFκB1 mRNAs, which is cleaved to form the mature NF-κB subunit p50, has been found to be directly modulated by miR-9. This subunit is significant in the transactivation of the p65 subunit allowing NF-κB to translocate to the nucleus. The direct interaction between miR-9 and NFκB1 was again established with luciferase reporter assays while overexpression of miR-9 in monocytes resulted in diminished NFκB1 protein levels. Unique to the MyD88-independent pathway, IKKε has also been identified as a target of miR-155. Predicted by the prediction algorithms, TargetScan and RNAhybrid, direct association was experimentally confirmed by luciferase reporter assays. Revealed by qRT-PCR, the mRNAs levels of IKKε were found to be altered by the transfection of human gastric epithelial GES-1 cells with miR-155 mimics. This indicates that miR-155 alters IKKε protein expression by mRNAs cleavage.

## miRNAs mitigation of TLR/IL-1 signaling regulators

The activity of signaling regulators must be closely monitored to ensure adequate activity and suppression of the TLR/IL-1 cascade when necessary. Therefore, the critical nature of these regulatory proteins perpetuates their potential for modulation by miRNAs. As mentioned previously, SOCS1 plays an integral role in regulating TLR/IL-1 signaling by targeting several proteins within the pathway. The ability of miRNAs to modulate SOCS1 expression would therefore have a significant effect on TLR/IL-1 signaling. In fact, several groups have reported that SOCS1 expression can be directly mediated by miR-155 [130-132]. Direct association between SOCS1 mRNAs and miR-155 was confirmed using luciferase reporter constructs containing the native 3’ UTR of SOCS1 mRNAs. Using the target prediction software PicTar, the miR-155 binding site sequence was identified and the native 3’ UTR of SOCS1 mRNAs was mutated to verify binding-site specificity. Also, an arbitrary miRNA not predicted to bind to SOCS1 was tested to verify miR-155 specificity [130,131]. This direct association was then further verified with a gel shift mobility assay that examined the ability of miR-155 to form duplexes with the predicted binding site sequence within the native 3’ UTR of SOCS1 mRNAs. Again, a mutated sequence was used to verify the binding site while anti-sense miR-155 was utilized as a positive control [131]. With association clearly demonstrated by the previous experiments, Western blot analysis was performed to verify that SOCS1 protein expression was mitigated. Transfection with miR-155 mimics or with miR-155-containing adenoviruses both impaired the protein expression of SOCS1 in a similar fashion to SOCS1 siRNAs [131,132]. These results were further supported by observations in miR-155 deficient mice, which showed elevated SOCS1 expression in T-cells [130]. Notably, SOCS1 mRNAs levels remained unaffected in the presence of miR-155 mimics, miR-155 inhibitors, or in miR-155-deficient mice. This indicates that SOCS1 expression is modulated by miR-155 via translational repression but not via mRNAs degradation.

miR-155 not only plays a critical role in SOCS1 expression but also has been confirmed in epithelial cells, macrophages, and myeloid cells to regulate SHIP1 expression [133-135]. Bioinformatics exploration with the target prediction software TargetScan revealed a highly conserved miR-155 binding site in SHIP1’s 3’UTR. In fact, this was the only highly conserved miRNAs binding site within the 3’UTR of SHIP1 [133]. Direct association was experimentally confirmed with a luciferase report assay where 293 T-cells were co-transfected with miR-155 mimics and wild-type SHIP1 3’UTR reporter plasmids. Those cells transfected with the mimics displayed impaired chemoluminescence in comparison to those transfected with the control miRNAs [134,135]. Predictably, cells transfected with scrambled SHIP1 3’UTR reporter plasmids showed no variability in chemoluminescence with either miR-155 mimics or controls [133,134]. In a separate study where cystic fibrosis IB3 epithelial cells have diminished levels of SHIP1 mRNAs compared to normal IB3 epithelial cells, treatment of fibrosis IB3 epithelial cells with miR-155 inhibitors led to increased luciferase activity associated with normal IB3 SHIP1 levels [135]. With miR-155:SHIP1 association confirmed, Western blots were performed to verify a change in SHIP1 protein levels. Utilizing miR-155-containing vectors it was demonstrated that SHIP1 expression was reduced 2.3 folds. Additionally, the use miR-155 mutant vectors showed no significant fold changes, verifying miR-155 specificity [133]. Furthermore, observation of miR-155-deficient macrophages revealed elevated SHIP-1 protein expression in comparison with wild-type controls [133,134]. RAW 264.7 cells transfected with wild-type miR-155 vectors led to an approximately 50% reduction in SHIP1 mRNAs when compared to the flag control. This effect was not seen in transfections with miR-155 mutant vectors [133]. These findings were supported by the data collected from a separate investigator conducting transfections with miR-155 mimics and inhibitors. Again, the expression of SHIP1 mRNAs was diminished by approximately 50% with miR-155 overexpression while inhibition of miR-155 function led to an equivalent increase [134]. Taken together, these dramatic changes in SHIP1 mRNAs indicate that miRNA-155 regulates SHIP1 expression by mRNAs degradation.

IL-10 is a prototypical anti-inflammatory cytokine which has been shown to affect IRAK4 and TRAF6 ubiquitination as described earlier. Utilizing three miRNAs target prediction programs, miR-106a was identified as having a binding-site in IL-10’s 3’UTR. Experiments conducted in B lymphoma lymphoblast-like Raji cells with miR-106a mimics resulted in a dose-dependent reduction of IL-10 protein in the culture supernatant. IL-10 mRNAs levels were also reduced in a dose-dependent manner when examined by qRT-PCR. The target site within the 3’UTR of IL-10 mRNAs was then confirmed in T cell leukemia Jurkat cells and Raji cells with luciferase reporter assays. These results indicate that IL-10 expression is capable of being transcriptionally regulated by miR-106a [136]. Mentioned previously, pro-inflammatory protein PDCD4 is capable of repressing IL-10 and therefore the repression of PDCD4 by miRNAs would in effect increase IL-10’s TLR/IL-1 regulatory activity. As it is, miR-21 has been found to target PDCD4. Transfecting RAW264.7 cells with miR-21 inhibitors mitigated the inhibitory effects of PDCD4 on IL-10 typically seen following LPS stimulation. Similar findings were found when competitive oligonucleotides that were specific to the miR-21 binding site in the PDCD4 3’UTR were used [137].

## miRNAs expression modulation by TLR/IL-1 signaling

As we have discussed, several miRNAs play an important role in regulating the TLR/IL-1 signaling pathway. Interestingly, recent findings have revealed that the activation and induction of this very important pathway can modulate the expression of several miRNAs [138]. Among them are some of the same miRNAs that regulate TLR/IL-1 signaling, indicating that these miRNAs participate in either positive or negative feedback mechanisms. The first miRNA identified to be modulated by TLR/IL-1 signaling was miR-146. Its expression was shown to be rapidly augmented following the exposure of human acute monocytic leukemia THP-1 cells to LPS. Further studies have shown that miR-146 can also be induced by ligands for TLR-2, -3, and −5 in macrophages, bone marrow-derived monocytes, and T-cells [139]. In addition, the expression of miR-146 was shown to be up-regulated by IL-1β [126]. Mechanistically, the ability of TLR/IL-1 signaling to induce miR-146 expression has been shown to be NF-κB-dependent. Taken into account that experiments with over-expressing miR-146 epithelial cells and fibroblasts resulted in a reduction of IL-1-induced cytokine expression, and that miR-146 is known to target the critical TLR/IL-1 signaling molecules IRAK1 and TRAF6, one can conclude that miR-146 is capable of mitigating a TLR/IL-1 inflammatory response in a negative feedback manner.

One of the most extensively characterized miRNAs, miR-155, has been proven to be induced by several TLR ligands in mouse bone marrow-derived macrophages. Specifically, ligands for TLR-2, -3, -4, and −9, were found to augment miR-155 expression [140]. Interestingly, this indicates that both the MyD88-dependent and MyD88-independent pathway can augment miR-155 expression. Mechanistically, both TLR/IL-1 downstream transcription factors, NF-κB and AP-1, have been shown to facilitate the transcription activation of the B-cell integration cluster (Bic), the gene that encodes for miRNA-155 [140,141]. This data clearly shows that miR-155 expression is augmented by TLR/IL-1 activity while direct miR-155 targeting of TLR/IL-1 signaling molecules MyD88, TAB2, and IKKε suggests that miR-155 can suppress TLR/IL-1 signaling by a negative feedback mechanism. Interestingly, miRNA-155 has also been credited with reciprocally facilitating TLR/IL-1 signaling. Experimentally, over-expression of miR-155 in the bone marrow compartment of adult mice led to myeloproliferation typically observed following LPS injection [142]. This may be explained by the fact that miR-155 is known to directly repress the activities of TLR/IL-1 signaling inhibitors SHIP1 and SOCS1. In this case, miR-155 would participate in a positive-feedback loop.

These differing results suggest several key points about miRNAs regulation. First, miRNAs activity and expression is heavily dependent on specific cell types; second, the preferential targeting of individual mRNA may be highly dependent on the expression of those mRNA or other unforeseen factors; and third, that seeming contradictive targeting may in fact be avoided by precisely orchestrated timing. Taking these considerations into account the targeting of SOCS1 and SHIP1 by miRNA-155 may initially be required to augment novel TLR/IL-1 signal transduction when an immune response is required. However, the perpetuation of this signal may need to be suppressed after a specific duration of inflammation initiation to prevent detrimental effects. Under these conditions the repression of adaptor molecules MyD88, TAB2, and IKKε would be required.

miR-9 has also been shown to be augmented by TLR/IL-1 activity in monocytes and granulocytes [143,144]. While mature miR-9 can be produced from three unique primary transcripts encoded by three distinct genes, LPS treatment only induced one of these primary transcripts, miR9-1. The promoter region of the chromosome 1 open reading frame 61 (C1orf61) locus, which generates this particular primary transcript, contains consensus binding sites for known LPS-sensitive transcription factors like NF-κB. This evidence suggests that rapid activation of the TLR/IL-1 signaling pathway leads to the induction of miR-9 through NF-κB-directed transcription of the miR9-1 transcript. In fact, the necessity of NF-κB in miR-9 induction was confirmed with the use of several different NF-κB inhibitors. Furthermore, inhibitors for p38 and JNK demonstrated no inhibitory effect on miR-9 induction following TLR/IL-1 signaling activation, further suggesting NF-κB’s involvement. Therefore, miR-9 can be described as a negative feedback regulator of TLR/IL-1 signaling since it targets the NF-κB subunit 50 as previously mentioned [143].

Another miRNAs shown to be elevated by TLR/IL-1 signaling in B-cells, cholangiocytes (epithelial cells of the bile duct), macrophages, and inflamed lung tissue is miR-21 [137,145-147]. This effect was abolished in immortalized, bone-marrow derived monocytes (BMDMs) deficient in MyD88 but was only slightly diminished in TRIF-deficient BMDMs. This suggests that the augmentation of miR-21’s expression is NF-κB-dependent. Further investigation revealed that the promoter of miRNA-21 contains a predicted NF-κB binding site. Its significance was confirmed in mouse embryonic fibroblasts that were deficient in the NF-κB p65 subunit. In these cells miRNA-21 levels could not be induced by LPS. Following LPS stimulation in RAW264.7 macrophages, the levels of miR-21 are not significantly induced until 4 hours and continue to increase until 24 hours. This observation is in accordance with the fact that PDCD4 expression immediately increases following LPS treatment but then is diminished at 4 hours and abolished at 24 hours [137]. Clearly, the expression of PDCD4 following LPS treatment is dependent on miR-21 expression. Furthermore, the reduction of PDCD4 by miR-21 explains the augmented levels of IL-10 observed after LPS treatment. The induced expression of miR-21 by TLR/IL-1 signaling results in a negative feedback loop by reducing PDCD4, causing greater IL-10 activity.

## Conclusion

The TLR/IL-1 signaling pathway is critical to proper immune functioning. Hyper-activation has been associated with several autoimmune and chronic pathologies while hypo-activation leaves the host highly susceptible to infection. Therefore, it is not surprising that this signaling pathway is under stringent control by phosphorylation, physical interactions, conformational changes, regulatory proteins, as well as ubiquitin and proteasomal degradation. We have also discussed the activity of miRNAs in further modulating this signaling system. Interestingly, many of these exact miRNAs are also induced by the activation of the TLR/IL-1 signaling cascade, indicating a mutual regulation between miRNAs and TLR/IL-1 pathway via a complex positive- and negative-feedback loop system [148]. The role of these participating miRNAs needs to be closely evaluated as they hold vast potential as novel therapeutics.

## Competing interests

The authors declare that they have no competing interests.

## Authors’ contributions

AV carried out the primary literature search and draft writing. HW and XFY provided field expertise, material input, and manuscript oversight. All authors read and approved the final manuscript.

## References

[B1] Finishing the euchromatic sequence of the human genome Nature 2004 431 931 945 15496913 10.1038/nature03001

[B2] Bartel DP MicroRNAs: target recognition and regulatory functions Cell 2009 136 215 233 19167326 10.1016/j.cell.2009.01.002 PMC3794896

[B3] Carthew RW Sontheimer EJ Origins and Mechanisms of miRNAs and siRNAs Cell 2009 136 642 655 19239886 10.1016/j.cell.2009.01.035 PMC2675692

[B4] Krol J Loedige I Filipowicz W The widespread regulation of microRNA biogenesis, function and decay Nature reviews 2010 11 597 610 10.1038/nrg2843 20661255

[B5] Voinnet O Origin, biogenesis, and activity of plant microRNAs Cell 2009 136 669 687 19239888 10.1016/j.cell.2009.01.046

[B6] Kim VN MicroRNA biogenesis: coordinated cropping and dicing Nat Rev Mol Cell Biol 2005 6 376 385 15852042 10.1038/nrm1644

[B7] Lee Y Jeon K Lee JT Kim S Kim VN MicroRNA maturation: stepwise processing and subcellular localization EMBO J 2002 21 4663 4670 12198168 10.1093/emboj/cdf476 PMC126204

[B8] Grimson A Farh KK Johnston WK Garrett-Engele P Lim LP Bartel DP MicroRNA targeting specificity in mammals: determinants beyond seed pairing Mol Cell 2007 27 91 105 17612493 10.1016/j.molcel.2007.06.017 PMC3800283

[B9] Brennecke J Stark A Russell RB Cohen SM Principles of microRNA-target recognition PLoS Biol 2005 3 e85 15723116 10.1371/journal.pbio.0030085 PMC1043860

[B10] Krek A Grun D Poy MN Wolf R Rosenberg L Epstein EJ MacMenamin P da Piedade I Gunsalus KC Stoffel M Rajewsky N Combinatorial microRNA target predictions Nat Genet 2005 37 495 500 15806104 10.1038/ng1536

[B11] Lewis BP Burge CB Bartel DP Conserved seed pairing, often flanked by adenosines, indicates that thousands of human genes are microRNA targets Cell 2005 120 15 20 15652477 10.1016/j.cell.2004.12.035

[B12] Lewis BP Shih IH Jones-Rhoades MW Bartel DP Burge CB Prediction of mammalian microRNA targets Cell 2003 115 787 798 14697198 10.1016/s0092-8674(03)01018-3

[B13] Doench JG Sharp PA Specificity of microRNA target selection in translational repression Genes Dev 2004 18 504 511 15014042 10.1101/gad.1184404 PMC374233

[B14] Corcoran DL Georgiev S Mukherjee N Gottwein E Skalsky RL Keene JD Ohler U PARalyzer: definition of RNA binding sites from PAR-CLIP short-read sequence data Genome Biol 2011 12 R79 21851591 10.1186/gb-2011-12-8-r79 PMC3302668

[B15] Nielsen CB Shomron N Sandberg R Hornstein E Kitzman J Burge CB Determinants of targeting by endogenous and exogenous microRNAs and siRNAs RNA 2007 13 1894 1910 (New York, NY) 17872505 10.1261/rna.768207 PMC2040081

[B16] Farh KK Grimson A Jan C Lewis BP Johnston WK Lim LP Burge CB Bartel DP The widespread impact of mammalian MicroRNAs on mRNA repression and evolution Science 2005 310 1817 1821 (New York, NY) 16308420 10.1126/science.1121158

[B17] Lim LP Lau NC Garrett-Engele P Grimson A Schelter JM Castle J Bartel DP Linsley PS Johnson JM Microarray analysis shows that some microRNAs downregulate large numbers of target mRNAs Nature 2005 433 769 773 15685193 10.1038/nature03315

[B18] Lai EC Tam B Rubin GM Pervasive regulation of Drosophila Notch target genes by GY-box-, Brd-box-, and K-box-class microRNAs Genes Dev 2005 19 1067 1080 15833912 10.1101/gad.1291905 PMC1091741

[B19] Saetrom P Heale BS Snove O Jr Aagaard L Alluin J Rossi JJ Distance constraints between microRNA target sites dictate efficacy and cooperativity Nucleic Acids Res 2007 35 2333 2342 17389647 10.1093/nar/gkm133 PMC1874663

[B20] Song JJ Smith SK Hannon GJ Joshua-Tor L Crystal structure of Argonaute and its implications for RISC slicer activity Science 2004 305 1434 1437 New York, NY 15284453 10.1126/science.1102514

[B21] Hutvagner G Simard MJ Argonaute proteins: key players in RNA silencing Nat Rev Mol Cell Biol 2008 9 22 32 18073770 10.1038/nrm2321

[B22] Liu J Carmell MA Rivas FV Marsden CG Thomson JM Song JJ Hammond SM Joshua-Tor L Hannon GJ Argonaute2 is the catalytic engine of mammalian RNAi Science 2004 305 1437 1441 New York, NY 15284456 10.1126/science.1102513

[B23] Meister G Landthaler M Patkaniowska A Dorsett Y Teng G Tuschl T Human Argonaute2 mediates RNA cleavage targeted by miRNAs and siRNAs Mol Cell 2004 15 185 197 15260970 10.1016/j.molcel.2004.07.007

[B24] Cerutti L Mian N Bateman A Domains in gene silencing and cell differentiation proteins: the novel PAZ domain and redefinition of the Piwi domain Trends Biochem Sci 2000 25 481 482 11050429 10.1016/s0968-0004(00)01641-8

[B25] Lingel A Simon B Izaurralde E Sattler M Structure and nucleic-acid binding of the Drosophila Argonaute 2 PAZ domain Nature 2003 426 465 469 14615801 10.1038/nature02123

[B26] Ma JB Ye K Patel DJ Structural basis for overhang-specific small interfering RNA recognition by the PAZ domain Nature 2004 429 318 322 15152257 10.1038/nature02519 PMC4700412

[B27] Song JJ Liu J Tolia NH Schneiderman J Smith SK Martienssen RA Hannon GJ Joshua-Tor L The crystal structure of the Argonaute2 PAZ domain reveals an RNA binding motif in RNAi effector complexes Nat Struct Biol 2003 10 1026 1032 14625589 10.1038/nsb1016

[B28] Yan KS Yan S Farooq A Han A Zeng L Zhou MM Structure and conserved RNA binding of the PAZ domain Nature 2003 426 468 474 14615802 10.1038/nature02129

[B29] Ma JB Yuan YR Meister G Pei Y Tuschl T Patel DJ Structural basis for 5'-end-specific recognition of guide RNA by the A. fulgidus Piwi protein Nature 2005 434 666 670 15800629 10.1038/nature03514 PMC4694588

[B30] Parker JS Roe SM Barford D Crystal structure of a PIWI protein suggests mechanisms for siRNA recognition and slicer activity EMBO J 2004 23 4727 4737 15565169 10.1038/sj.emboj.7600488 PMC535097

[B31] Yuan YR Pei Y Ma JB Kuryavyi V Zhadina M Meister G Chen HY Dauter Z Tuschl T Patel DJ Crystal structure of A. aeolicus argonaute, a site-specific DNA-guided endoribonuclease, provides insights into RISC-mediated mRNA cleavage Mol Cell 2005 19 405 419 16061186 10.1016/j.molcel.2005.07.011 PMC4689305

[B32] Guo HS Xie Q Fei JF Chua NH MicroRNA directs mRNA cleavage of the transcription factor NAC1 to downregulate auxin signals for arabidopsis lateral root development Plant Cell 2005 17 1376 1386 15829603 10.1105/tpc.105.030841 PMC1091761

[B33] Mallory AC Reinhart BJ Jones-Rhoades MW Tang G Zamore PD Barton MK Bartel DP MicroRNA control of PHABULOSA in leaf development: importance of pairing to the microRNA 5' region EMBO J 2004 23 3356 3364 15282547 10.1038/sj.emboj.7600340 PMC514513

[B34] Yekta S Shih IH Bartel DP MicroRNA-directed cleavage of HOXB8 mRNA Science 2004 304 594 596 (New York, NY 15105502 10.1126/science.1097434

[B35] Chen X A microRNA as a translational repressor of APETALA2 in Arabidopsis flower development Science 2004 303 2022 2025 New York, NY 12893888 10.1126/science.1088060 PMC5127708

[B36] Parker R Song H The enzymes and control of eukaryotic mRNA turnover Nat Struct Mol Biol 2004 11 121 127 14749774 10.1038/nsmb724

[B37] Valencia-Sanchez MA Liu J Hannon GJ Parker R Control of translation and mRNA degradation by miRNAs and siRNAs Genes Dev 2006 20 515 524 16510870 10.1101/gad.1399806

[B38] Brennecke J Hipfner DR Stark A Russell RB Cohen SM Bantam encodes a developmentally regulated microRNA that controls cell proliferation and regulates the proapoptotic gene hid in Drosophila Cell 2003 113 25 36 12679032 10.1016/s0092-8674(03)00231-9

[B39] Cimmino A Calin GA Fabbri M Iorio MV Ferracin M Shimizu M Wojcik SE Aqeilan RI Zupo S Dono M miR-15 and miR-16 induce apoptosis by targeting BCL2 Proc Natl Acad Sci USA 2005 102 13944 13949 16166262 10.1073/pnas.0506654102 PMC1236577

[B40] Lee RC Feinbaum RL Ambros V The C. elegans heterochronic gene lin-4 encodes small RNAs with antisense complementarity to lin-14 Cell 1993 75 843 854 8252621 10.1016/0092-8674(93)90529-y

[B41] Poy MN Eliasson L Krutzfeldt J Kuwajima S Ma X Macdonald PE Pfeffer S Tuschl T Rajewsky N Rorsman P Stoffel M A pancreatic islet-specific microRNA regulates insulin secretion Nature 2004 432 226 230 15538371 10.1038/nature03076

[B42] Wightman B Ha I Ruvkun G Posttranscriptional regulation of the heterochronic gene lin-14 by lin-4 mediates temporal pattern formation in C. elegans Cell 1993 75 855 862 8252622 10.1016/0092-8674(93)90530-4

[B43] Bhattacharyya SN Habermacher R Martine U Closs EI Filipowicz W Relief of microRNA-mediated translational repression in human cells subjected to stress Cell 2006 125 1111 1124 16777601 10.1016/j.cell.2006.04.031

[B44] Chendrimada TP Finn KJ Ji X Baillat D Gregory RI Liebhaber SA Pasquinelli AE Shiekhattar R MicroRNA silencing through RISC recruitment of eIF6 Nature 2007 447 823 828 17507929 10.1038/nature05841

[B45] Ding XC Grosshans H Repression of C. elegans microRNA targets at the initiation level of translation requires GW182 proteins EMBO J 2009 28 213 222 19131968 10.1038/emboj.2008.275 PMC2637332

[B46] Gu S Jin L Zhang F Sarnow P Kay MA Biological basis for restriction of microRNA targets to the 3' untranslated region in mammalian mRNAs Nat Struct Mol Biol 2009 16 144 150 19182800 10.1038/nsmb.1552 PMC2713750

[B47] Mathonnet G Fabian MR Svitkin YV Parsyan A Huck L Murata T Biffo S Merrick WC Darzynkiewicz E Pillai RS MicroRNA inhibition of translation initiation in vitro by targeting the cap-binding complex eIF4F Science 2007 317 1764 1767 New York, NY 17656684 10.1126/science.1146067

[B48] Nottrott S Simard MJ Richter JD Human let-7a miRNA blocks protein production on actively translating polyribosomes Nat Struct Mol Biol 2006 13 1108 1114 17128272 10.1038/nsmb1173

[B49] Petersen CP Bordeleau ME Pelletier J Sharp PA Short RNAs repress translation after initiation in mammalian cells Mol Cell 2006 21 533 542 16483934 10.1016/j.molcel.2006.01.031

[B50] Pillai RS Bhattacharyya SN Artus CG Zoller T Cougot N Basyuk E Bertrand E Filipowicz W Inhibition of translational initiation by Let-7 MicroRNA in human cells Science 2005 309 1573 1576 New York, NY 16081698 10.1126/science.1115079

[B51] Ding L Han M GW182 family proteins are crucial for microRNA-mediated gene silencing Trends Cell Biol 2007 17 411 416 17766119 10.1016/j.tcb.2007.06.003

[B52] Eulalio A Behm-Ansmant I Schweizer D Izaurralde E P-body formation is a consequence, not the cause, of RNA-mediated gene silencing Mol Cell Biol 2007 27 3970 3981 17403906 10.1128/MCB.00128-07 PMC1900022

[B53] Cannell IG Kong YW Bushell M How do microRNAs regulate gene expression? Biochem Soc Trans 2008 36 1224 1231 19021530 10.1042/BST0361224

[B54] Kiriakidou M Tan GS Lamprinaki S De Planell-Saguer M Nelson PT Mourelatos Z An mRNA m7G cap binding-like motif within human Ago2 represses translation Cell 2007 129 1141 1151 17524464 10.1016/j.cell.2007.05.016

[B55] Kim J Krichevsky A Grad Y Hayes GD Kosik KS Church GM Ruvkun G Identification of many microRNAs that copurify with polyribosomes in mammalian neurons Proc Natl Acad Sci USA 2004 101 360 365 14691248 10.1073/pnas.2333854100 PMC314190

[B56] Kong YW Cannell IG de Moor CH Hill K Garside PG Hamilton TL Meijer HA Dobbyn HC Stoneley M Spriggs KA The mechanism of micro-RNA-mediated translation repression is determined by the promoter of the target gene Proc Natl Acad Sci USA 2008 105 8866 8871 18579786 10.1073/pnas.0800650105 PMC2449332

[B57] Maroney PA Yu Y Fisher J Nilsen TW Evidence that microRNAs are associated with translating messenger RNAs in human cells Nat Struct Mol Biol 2006 13 1102 1107 17128271 10.1038/nsmb1174

[B58] Thermann R Hentze MW Drosophila miR2 induces pseudo-polysomes and inhibits translation initiation Nature 2007 447 875 878 17507927 10.1038/nature05878

[B59] Slack JL Schooley K Bonnert TP Mitcham JL Qwarnstrom EE Sims JE Dower SK Identification of two major sites in the type I interleukin-1 receptor cytoplasmic region responsible for coupling to pro-inflammatory signaling pathways J Biol Chem 2000 275 4670 4678 10671496 10.1074/jbc.275.7.4670

[B60] Janeway CA Jr Medzhitov R Innate immune recognition Annu Rev Immunol 2002 20 197 216 11861602 10.1146/annurev.immunol.20.083001.084359

[B61] Smiley ST King JA Hancock WW Fibrinogen stimulates macrophage chemokine secretion through toll-like receptor 4 J Immunol 2001 167 2887 2894 11509636 10.4049/jimmunol.167.5.2887

[B62] Rassa JC Meyers JL Zhang Y Kudaravalli R Ross SR Murine retroviruses activate B cells via interaction with toll-like receptor 4 Proc Natl Acad Sci USA 2002 99 2281 2286 11854525 10.1073/pnas.042355399 PMC122356

[B63] Kurt-Jones EA Popova L Kwinn L Haynes LM Jones LP Tripp RA Walsh EE Freeman MW Golenbock DT Anderson LJ Finberg RW Pattern recognition receptors TLR4 and CD14 mediate response to respiratory syncytial virus Nat Immunol 2000 1 398 401 11062499 10.1038/80833

[B64] Kawasaki K Akashi S Shimazu R Yoshida T Miyake K Nishijima M Mouse toll-like receptor 4.MD-2 complex mediates lipopolysaccharide-mimetic signal transduction by Taxol J Biol Chem 2000 275 2251 2254 10644670 10.1074/jbc.275.4.2251

[B65] Bulut Y Faure E Thomas L Karahashi H Michelsen KS Equils O Morrison SG Morrison RP Arditi M Chlamydial heat shock protein 60 activates macrophages and endothelial cells through Toll-like receptor 4 and MD2 in a MyD88-dependent pathway J Immunol 2002 168 1435 1440 11801686 10.4049/jimmunol.168.3.1435

[B66] Ohashi K Burkart V Flohe S Kolb H Cutting edge: heat shock protein 60 is a putative endogenous ligand of the toll-like receptor-4 complex J Immunol 2000 164 558 561 10623794 10.4049/jimmunol.164.2.558

[B67] Poltorak A He X Smirnova I Liu MY Van Huffel C Du X Birdwell D Alejos E Silva M Galanos C Defective LPS signaling in C3H/HeJ and C57BL/10ScCr mice: mutations in Tlr4 gene Science 1998 282 2085 2088 (New York, NY 9851930 10.1126/science.282.5396.2085

[B68] Alexopoulou L Holt AC Medzhitov R Flavell RA Recognition of double-stranded RNA and activation of NF-kappaB by Toll-like receptor 3 Nature 2001 413 732 738 11607032 10.1038/35099560

[B69] Ahmad-Nejad P Hacker H Rutz M Bauer S Vabulas RM Wagner H Bacterial CpG-DNA and lipopolysaccharides activate Toll-like receptors at distinct cellular compartments Eur J Immunol 2002 32 1958 1968 12115616 10.1002/1521-4141(200207)32:7<1958::AID-IMMU1958>3.0.CO;2-U

[B70] Heil F Ahmad-Nejad P Hemmi H Hochrein H Ampenberger F Gellert T Dietrich H Lipford G Takeda K Akira S The Toll-like receptor 7 (TLR7)-specific stimulus loxoribine uncovers a strong relationship within the TLR7, 8 and 9 subfamily Eur J Immunol 2003 33 2987 2997 14579267 10.1002/eji.200324238

[B71] Matsumoto M Funami K Tanabe M Oshiumi H Shingai M Seto Y Yamamoto A Seya T Subcellular localization of Toll-like receptor 3 in human dendritic cells J Immunol 2003 171 3154 3162 12960343 10.4049/jimmunol.171.6.3154

[B72] Atkins E Fever: the old and the new J Infect Dis 1984 149 339 348 6232325 10.1093/infdis/149.3.339

[B73] Dinarello CA The biological properties of interleukin-1 Eur Cytokine Netw 1994 5 517 531 7727685

[B74] Akira S Takeda K Toll-like receptor signalling Nature reviews 2004 4 499 511 10.1038/nri1391 15229469

[B75] O'Neill LA Bowie AG The family of five: TIR-domain-containing adaptors in Toll-like receptor signalling Nature reviews 2007 7 353 364 10.1038/nri2079 17457343

[B76] Burns K Martinon F Esslinger C Pahl H Schneider P Bodmer JL Di Marco F French L Tschopp J MyD88, an adapter protein involved in interleukin-1 signaling J Biol Chem 1998 273 12203 12209 9575168 10.1074/jbc.273.20.12203

[B77] Janssens S Beyaert R Functional diversity and regulation of different interleukin-1 receptor-associated kinase (IRAK) family members Mol Cell 2003 11 293 302 12620219 10.1016/s1097-2765(03)00053-4

[B78] Wesche H Gao X Li X Kirschning CJ Stark GR Cao Z IRAK-M is a novel member of the Pelle/interleukin-1 receptor-associated kinase (IRAK) family J Biol Chem 1999 274 19403 19410 10383454 10.1074/jbc.274.27.19403

[B79] Li X Commane M Burns C Vithalani K Cao Z Stark GR Mutant cells that do not respond to interleukin-1 (IL-1) reveal a novel role for IL-1 receptor-associated kinase Mol Cell Biol 1999 19 4643 4652 10373513 10.1128/mcb.19.7.4643 PMC84262

[B80] Kanakaraj P Schafer PH Cavender DE Wu Y Ngo K Grealish PF Wadsworth SA Peterson PA Siekierka JJ Harris CA Fung-Leung WP Interleukin (IL)-1 receptor-associated kinase (IRAK) requirement for optimal induction of multiple IL-1 signaling pathways and IL-6 production J Exp Med 1998 187 2073 2079 9625767 10.1084/jem.187.12.2073 PMC2212370

[B81] Thomas JA Allen JL Tsen M Dubnicoff T Danao J Liao XC Cao Z Wasserman SA Impaired cytokine signaling in mice lacking the IL-1 receptor-associated kinase J Immunol 1999 163 978 984 10395695

[B82] Suzuki N Suzuki S Duncan GS Millar DG Wada T Mirtsos C Takada H Wakeham A Itie A Li S Severe impairment of interleukin-1 and Toll-like receptor signalling in mice lacking IRAK-4 Nature 2002 416 750 756 11923871 10.1038/nature736

[B83] Li S Strelow A Fontana EJ Wesche H IRAK-4: a novel member of the IRAK family with the properties of an IRAK-kinase Proc Natl Acad Sci USA 2002 99 5567 5572 11960013 10.1073/pnas.082100399 PMC122810

[B84] Cao Z Xiong J Takeuchi M Kurama T Goeddel DV TRAF6 is a signal transducer for interleukin-1 Nature 1996 383 443 446 8837778 10.1038/383443a0

[B85] Bradley JR Pober JS Tumor necrosis factor receptor-associated factors (TRAFs) Oncogene 2001 20 6482 6491 11607847 10.1038/sj.onc.1204788

[B86] Ye H Arron JR Lamothe B Cirilli M Kobayashi T Shevde NK Segal D Dzivenu OK Vologodskaia M Yim M Distinct molecular mechanism for initiating TRAF6 signalling Nature 2002 418 443 447 12140561 10.1038/nature00888

[B87] Takaesu G Surabhi RM Park KJ Ninomiya-Tsuji J Matsumoto K Gaynor RB TAK1 is critical for IkappaB kinase-mediated activation of the NF-kappaB pathway J Mol Biol 2003 326 105 115 12547194 10.1016/s0022-2836(02)01404-3

[B88] Shibuya H Yamaguchi K Shirakabe K Tonegawa A Gotoh Y Ueno N Irie K Nishida E Matsumoto K TAB1: an activator of the TAK1 MAPKKK in TGF-beta signal transduction Science 1996 272 1179 1182 New York, NY 8638164 10.1126/science.272.5265.1179

[B89] Takaesu G Kishida S Hiyama A Yamaguchi K Shibuya H Irie K Ninomiya-Tsuji J Matsumoto K TAB2, a novel adaptor protein, mediates activation of TAK1 MAPKKK by linking TAK1 to TRAF6 in the IL-1 signal transduction pathway Mol Cell 2000 5 649 658 10882101 10.1016/s1097-2765(00)80244-0

[B90] Sanjo H Takeda K Tsujimura T Ninomiya-Tsuji J Matsumoto K Akira S TAB2 is essential for prevention of apoptosis in fetal liver but not for interleukin-1 signaling Mol Cell Biol 2003 23 1231 1238 12556483 10.1128/MCB.23.4.1231-1238.2003 PMC141141

[B91] Ishitani T Takaesu G Ninomiya-Tsuji J Shibuya H Gaynor RB Matsumoto K Role of the TAB2-related protein TAB3 in IL-1 and TNF signaling EMBO J 2003 22 6277 6288 14633987 10.1093/emboj/cdg605 PMC291846

[B92] Takeda K Kaisho T Akira S Toll-like receptors Annu Rev Immunol 2003 21 335 376 12524386 10.1146/annurev.immunol.21.120601.141126

[B93] Medzhitov R Toll-like receptors and innate immunity Nature reviews 2001 1 135 145 10.1038/35100529 11905821

[B94] Barton GM Medzhitov R Toll-like receptor signaling pathways Science 2003 300 1524 1525 New York, NY 12791976 10.1126/science.1085536

[B95] Moriguchi T Kuroyanagi N Yamaguchi K Gotoh Y Irie K Kano T Shirakabe K Muro Y Shibuya H Matsumoto K A novel kinase cascade mediated by mitogen-activated protein kinase kinase 6 and MKK3 J Biol Chem 1996 271 13675 13679 8663074 10.1074/jbc.271.23.13675

[B96] Mochida Y Takeda K Saitoh M Nishitoh H Amagasa T Ninomiya-Tsuji J Matsumoto K Ichijo H ASK1 inhibits interleukin-1-induced NF-kappa B activity through disruption of TRAF6-TAK1 interaction J Biol Chem 2000 275 32747 32752 10921914 10.1074/jbc.M003042200

[B97] Matsuzawa A Saegusa K Noguchi T Sadamitsu C Nishitoh H Nagai S Koyasu S Matsumoto K Takeda K Ichijo H ROS-dependent activation of the TRAF6-ASK1-p38 pathway is selectively required for TLR4-mediated innate immunity Nat Immunol 2005 6 587 592 15864310 10.1038/ni1200

[B98] Ichijo H Nishida E Irie K ten Dijke P Saitoh M Moriguchi T Takagi M Matsumoto K Miyazono K Gotoh Y Induction of apoptosis by ASK1, a mammalian MAPKKK that activates SAPK/JNK and p38 signaling pathways Science 1997 275 90 94 New York, NY 8974401 10.1126/science.275.5296.90

[B99] Yamamoto M Sato S Hemmi H Hoshino K Kaisho T Sanjo H Takeuchi O Sugiyama M Okabe M Takeda K Akira S Role of adaptor TRIF in the MyD88-independent toll-like receptor signaling pathway Science 2003 301 640 643 New York, NY 12855817 10.1126/science.1087262

[B100] Fitzgerald KA McWhirter SM Faia KL Rowe DC Latz E Golenbock DT Coyle AJ Liao SM Maniatis T IKKepsilon and TBK1 are essential components of the IRF3 signaling pathway Nat Immunol 2003 4 491 496 12692549 10.1038/ni921

[B101] Sharma S TenOever BR Grandvaux N Zhou GP Lin R Hiscott J Triggering the interferon antiviral response through an IKK-related pathway Science 2003 300 1148 1151 New York, NY 12702806 10.1126/science.1081315

[B102] Shimada T Kawai T Takeda K Matsumoto M Inoue J Tatsumi Y Kanamaru A Akira S IKK-i, a novel lipopolysaccharide-inducible kinase that is related to IkappaB kinases Int Immunol 1999 11 1357 1362 10421793 10.1093/intimm/11.8.1357

[B103] Pomerantz JL Baltimore D NF-kappaB activation by a signaling complex containing TRAF2, TANK and TBK1, a novel IKK-related kinase EMBO J 1999 18 6694 6704 10581243 10.1093/emboj/18.23.6694 PMC1171732

[B104] Peters RT Liao SM Maniatis T IKKepsilon is part of a novel PMA-inducible IkappaB kinase complex Mol Cell 2000 5 513 522 10882136 10.1016/s1097-2765(00)80445-1

[B105] Nomura F Kawai T Nakanishi K Akira S NF-kappaB activation through IKK-i-dependent I-TRAF/TANK phosphorylation Genes Cells 2000 5 191 202 10759890 10.1046/j.1365-2443.2000.00315.x

[B106] Yamamoto M Sato S Hemmi H Uematsu S Hoshino K Kaisho T Takeuchi O Takeda K Akira S TRAM is specifically involved in the Toll-like receptor 4-mediated MyD88-independent signaling pathway Nat Immunol 2003 4 1144 1150 14556004 10.1038/ni986

[B107] Kimura A Naka T Muta T Takeuchi O Akira S Kawase I Kishimoto T Suppressor of cytokine signaling-1 selectively inhibits LPS-induced IL-6 production by regulating JAK-STAT Proc Natl Acad Sci USA 2005 102 17089 17094 16287972 10.1073/pnas.0508517102 PMC1288004

[B108] Naka T Fujimoto M Tsutsui H Yoshimura A Negative regulation of cytokine and TLR signalings by SOCS and others Adv Immunol 2005 87 61 122 16102572 10.1016/S0065-2776(05)87003-8

[B109] Nakagawa R Naka T Tsutsui H Fujimoto M Kimura A Abe T Seki E Sato S Takeuchi O Takeda K SOCS-1 participates in negative regulation of LPS responses Immunity 2002 17 677 687 12433373 10.1016/s1074-7613(02)00449-1

[B110] Kinjyo I Hanada T Inagaki-Ohara K Mori H Aki D Ohishi M Yoshida H Kubo M Yoshimura A SOCS1/JAB is a negative regulator of LPS-induced macrophage activation Immunity 2002 17 583 591 12433365 10.1016/s1074-7613(02)00446-6

[B111] Hanada T Tanaka K Matsumura Y Yamauchi M Nishinakamura H Aburatani H Mashima R Kubo M Kobayashi T Yoshimura A Induction of hyper Th1 cell-type immune responses by dendritic cells lacking the suppressor of cytokine signaling-1 gene J Immunol 2005 174 4325 4332 15778397 10.4049/jimmunol.174.7.4325

[B112] Chinen T Kobayashi T Ogata H Takaesu G Takaki H Hashimoto M Yagita H Nawata H Yoshimura A Suppressor of cytokine signaling-1 regulates inflammatory bowel disease in which both IFNgamma and IL-4 are involved Gastroenterology 2006 130 373 388 16472593 10.1053/j.gastro.2005.10.051

[B113] Gingras S Parganas E de Pauw A Ihle JN Murray PJ Re-examination of the role of suppressor of cytokine signaling 1 (SOCS1) in the regulation of toll-like receptor signaling J Biol Chem 2004 279 54702 54707 15491990 10.1074/jbc.M411043200

[B114] Waiboci LW Ahmed CM Mujtaba MG Flowers LO Martin JP Haider MI Johnson HM Both the suppressor of cytokine signaling 1 (SOCS-1) kinase inhibitory region and SOCS-1 mimetic bind to JAK2 autophosphorylation site: implications for the development of a SOCS-1 antagonist J Immunol 2007 178 5058 5068 17404288 10.4049/jimmunol.178.8.5058

[B115] Mansell A Smith R Doyle SL Gray P Fenner JE Crack PJ Nicholson SE Hilton DJ O'Neill LA Hertzog PJ Suppressor of cytokine signaling 1 negatively regulates Toll-like receptor signaling by mediating Mal degradation Nat Immunol 2006 7 148 155 16415872 10.1038/ni1299

[B116] He Y Zhang W Zhang R Zhang H Min W SOCS1 inhibits tumor necrosis factor-induced activation of ASK1-JNK inflammatory signaling by mediating ASK1 degradation J Biol Chem 2006 281 5559 5566 16407264 10.1074/jbc.M512338200

[B117] Neel BG Gu H Pao L The 'Shp'ing news: SH2 domain-containing tyrosine phosphatases in cell signaling Trends Biochem Sci 2003 28 284 293 12826400 10.1016/S0968-0004(03)00091-4

[B118] An H Xu H Zhang M Zhou J Feng T Qian C Qi R Cao X Src homology 2 domain-containing inositol-5-phosphatase 1 (SHIP1) negatively regulates TLR4-mediated LPS response primarily through a phosphatase activity- and PI-3K-independent mechanism Blood 2005 105 4685 4692 15701712 10.1182/blood-2005-01-0191

[B119] Gabhann JN Higgs R Brennan K Thomas W Damen JE Ben Larbi N Krystal G Jefferies CA Absence of SHIP-1 results in constitutive phosphorylation of tank-binding kinase 1 and enhanced TLR3-dependent IFN-beta production J Immunol 2010 184 2314 2320 20100929 10.4049/jimmunol.0902589

[B120] Sly LM Rauh MJ Kalesnikoff J Buchse T Krystal G SHIP, SHIP2, and PTEN activities are regulated in vivo by modulation of their protein levels: SHIP is up-regulated in macrophages and mast cells by lipopolysaccharide Exp Hematol 2003 31 1170 1181 14662322 10.1016/j.exphem.2003.09.011

[B121] Chang J Kunkel SL Chang CH Negative regulation of MyD88-dependent signaling by IL-10 in dendritic cells Proc Natl Acad Sci USA 2009 106 18327 18332 19815506 10.1073/pnas.0905815106 PMC2775313

[B122] Androulidaki A Iliopoulos D Arranz A Doxaki C Schworer S Zacharioudaki V Margioris AN Tsichlis PN Tsatsanis C The kinase Akt1 controls macrophage response to lipopolysaccharide by regulating microRNAs Immunity 2009 31 220 231 19699171 10.1016/j.immuni.2009.06.024 PMC2865583

[B123] Chen XM Splinter PL O'Hara SP LaRusso NF A cellular micro-RNA, let-7i, regulates Toll-like receptor 4 expression and contributes to cholangiocyte immune responses against Cryptosporidium parvum infection J Biol Chem 2007 282 28929 28938 17660297 10.1074/jbc.M702633200 PMC2194650

[B124] Benakanakere MR Li Q Eskan MA Singh AV Zhao J Galicia JC Stathopoulou P Knudsen TB Kinane DF Modulation of TLR2 protein expression by miR-105 in human oral keratinocytes J Biol Chem 2009 284 23107 23115 19509287 10.1074/jbc.M109.013862 PMC2755716

[B125] Tang B Xiao B Liu Z Li N Zhu ED Li BS Xie QH Zhuang Y Zou QM Mao XH Identification of MyD88 as a novel target of miR-155, involved in negative regulation of Helicobacter pylori-induced inflammation FEBS Lett 2010 584 1481 1486 20219467 10.1016/j.febslet.2010.02.063

[B126] Taganov KD Boldin MP Chang KJ Baltimore D NF-kappaB-dependent induction of microRNA miR-146, an inhibitor targeted to signaling proteins of innate immune responses Proc Natl Acad Sci USA 2006 103 12481 12486 16885212 10.1073/pnas.0605298103 PMC1567904

[B127] Hou J Wang P Lin L Liu X Ma F An H Wang Z Cao X MicroRNA-146a feedback inhibits RIG-I-dependent Type I IFN production in macrophages by targeting TRAF6, IRAK1, and IRAK2 J Immunol 2009 183 2150 2158 19596990 10.4049/jimmunol.0900707

[B128] Fang Y Shi C Manduchi E Civelek M Davies PF MicroRNA-10a regulation of proinflammatory phenotype in athero-susceptible endothelium in vivo and in vitro Proc Natl Acad Sci USA 2010 107 13450–13455 20624982 10.1073/pnas.1002120107 PMC2922125

[B129] Imaizumi T Tanaka H Tajima A Yokono Y Matsumiya T Yoshida H Tsuruga K Aizawa-Yashiro T Hayakari R Inoue I IFN-gamma and TNF-alpha synergistically induce microRNA-155 which regulates TAB2/IP-10 expression in human mesangial cells Am J Nephrol 2010 32 462 468 20948191 10.1159/000321365

[B130] Lu LF Thai TH Calado DP Chaudhry A Kubo M Tanaka K Loeb GB Lee H Yoshimura A Rajewsky K Rudensky AY Foxp3-dependent microRNA155 confers competitive fitness to regulatory T cells by targeting SOCS1 protein Immunity 2009 30 80 91 19144316 10.1016/j.immuni.2008.11.010 PMC2654249

[B131] Jiang S Zhang HW Lu MH He XH Li Y Gu H Liu MF Wang ED MicroRNA-155 functions as an OncomiR in breast cancer by targeting the suppressor of cytokine signaling 1 gene Cancer Res 2010 70 3119 3127 20354188 10.1158/0008-5472.CAN-09-4250

[B132] Wang P Hou J Lin L Wang C Liu X Li D Ma F Wang Z Cao X Inducible microRNA-155 feedback promotes type I IFN signaling in antiviral innate immunity by targeting suppressor of cytokine signaling 1 J Immunol 2010 185 6226 6233 20937844 10.4049/jimmunol.1000491

[B133] O'Connell RM Chaudhuri AA Rao DS Baltimore D Inositol phosphatase SHIP1 is a primary target of miR-155 Proc Natl Acad Sci USA 2009 106 7113 7118 19359473 10.1073/pnas.0902636106 PMC2678424

[B134] Kurowska-Stolarska M Alivernini S Ballantine LE Asquith DL Millar NL Gilchrist DS Reilly J Ierna M Fraser AR Stolarski B MicroRNA-155 as a proinflammatory regulator in clinical and experimental arthritis Proc Natl Acad Sci USA 2011 108 11193 11198 21690378 10.1073/pnas.1019536108 PMC3131377

[B135] Bhattacharyya S Balakathiresan NS Dalgard C Gutti U Armistead D Jozwik C Srivastava M Pollard HB Biswas R Elevated miR-155 promotes inflammation in cystic fibrosis by driving hyperexpression of interleukin-8 J Biol Chem 2011 286 11604 11615 21282106 10.1074/jbc.M110.198390 PMC3064214

[B136] Sharma A Kumar M Aich J Hariharan M Brahmachari SK Agrawal A Ghosh B Posttranscriptional regulation of interleukin-10 expression by hsa-miR-106a Proc Natl Acad Sci USA 2009 106 5761 5766 19307576 10.1073/pnas.0808743106 PMC2659714

[B137] Sheedy FJ Palsson-McDermott E Hennessy EJ Martin C O'Leary JJ Ruan Q Johnson DS Chen Y O'Neill LA Negative regulation of TLR4 via targeting of the proinflammatory tumor suppressor PDCD4 by the microRNA miR-21 Nat Immunol 2009 11 141 147 19946272 10.1038/ni.1828

[B138] Nahid MA Satoh M Chan EK MicroRNA in TLR signaling and endotoxin tolerance Cell Mol Immunol 2011 8 388 403 21822296 10.1038/cmi.2011.26 PMC3618661

[B139] O'Neill LA Sheedy FJ McCoy CE MicroRNAs: the fine-tuners of Toll-like receptor signalling Nature reviews 2011 11 163 175 10.1038/nri2957 21331081

[B140] O'Connell RM Taganov KD Boldin MP Cheng G Baltimore D MicroRNA-155 is induced during the macrophage inflammatory response Proc Natl Acad Sci USA 2007 104 1604 1609 17242365 10.1073/pnas.0610731104 PMC1780072

[B141] Thai TH Calado DP Casola S Ansel KM Xiao C Xue Y Murphy A Frendewey D Valenzuela D Kutok JL Regulation of the germinal center response by microRNA-155 Science 2007 316 604 608 New York, NY 17463289 10.1126/science.1141229

[B142] O'Connell RM Rao DS Chaudhuri AA Boldin MP Taganov KD Nicoll J Paquette RL Baltimore D Sustained expression of microRNA-155 in hematopoietic stem cells causes a myeloproliferative disorder J Exp Med 2008 205 585 594 18299402 10.1084/jem.20072108 PMC2275382

[B143] Bazzoni F Rossato M Fabbri M Gaudiosi D Mirolo M Mori L Tamassia N Mantovani A Cassatella MA Locati M Induction and regulatory function of miR-9 in human monocytes and neutrophils exposed to proinflammatory signals Proc Natl Acad Sci USA 2009 106 5282 5287 19289835 10.1073/pnas.0810909106 PMC2664036

[B144] Ma X Becker Buscaglia LE Barker JR Li Y MicroRNAs in NF-kappaB signaling J Mol Cell Biol 2011 3 159 166 21502305 10.1093/jmcb/mjr007 PMC3104013

[B145] Gao W Shen H Liu L Xu J Xu J Shu Y MiR-21 overexpression in human primary squamous cell lung carcinoma is associated with poor patient prognosis J Cancer Res Clin Oncol 2011 137 557 566 20508945 10.1007/s00432-010-0918-4 PMC11828261

[B146] Medina PP Nolde M Slack FJ OncomiR addiction in an in vivo model of microRNA-21-induced pre-B-cell lymphoma Nature 2010 467 86 90 20693987 10.1038/nature09284

[B147] Zhou R Hu G Liu J Gong AY Drescher KM Chen XM NF-kappaB p65-dependent transactivation of miRNA genes following Cryptosporidium parvum infection stimulates epithelial cell immune responses PLoS Pathog 2009 5 e1000681 19997496 10.1371/journal.ppat.1000681 PMC2778997

[B148] Pasquinelli AE MicroRNAs and their targets: recognition, regulation and an emerging reciprocal relationship Nature reviews 13 271 282 10.1038/nrg3162 22411466

